# A Lightweight Multi-Scale Convolutional Neural Network for P300 Decoding: Analysis of Training Strategies and Uncovering of Network Decision

**DOI:** 10.3389/fnhum.2021.655840

**Published:** 2021-07-08

**Authors:** Davide Borra, Silvia Fantozzi, Elisa Magosso

**Affiliations:** ^1^Department of Electrical, Electronic and Information Engineering “Guglielmo Marconi” (DEI), University of Bologna, Cesena, Italy; ^2^Interdepartmental Center for Industrial Research on Health Sciences & Technologies, University of Bologna, Bologna, Italy; ^3^Alma Mater Research Institute for Human-Centered Artificial Intelligence, University of Bologna, Bologna, Italy

**Keywords:** electroencephalography, P300, convolutional neural networks, transfer learning, decision explanation, brain-computer interfaces

## Abstract

Convolutional neural networks (CNNs), which automatically learn features from raw data to approximate functions, are being increasingly applied to the end-to-end analysis of electroencephalographic (EEG) signals, especially for decoding brain states in brain-computer interfaces (BCIs). Nevertheless, CNNs introduce a large number of trainable parameters, may require long training times, and lack in interpretability of learned features. The aim of this study is to propose a CNN design for P300 decoding with emphasis on its lightweight design while guaranteeing high performance, on the effects of different training strategies, and on the use of *post-hoc* techniques to explain network decisions. The proposed design, named MS-EEGNet, learned temporal features in two different timescales (i.e., multi-scale, MS) in an efficient and optimized (in terms of trainable parameters) way, and was validated on three P300 datasets. The CNN was trained using different strategies (within-participant and within-session, within-participant and cross-session, leave-one-subject-out, transfer learning) and was compared with several state-of-the-art (SOA) algorithms. Furthermore, variants of the baseline MS-EEGNet were analyzed to evaluate the impact of different hyper-parameters on performance. Lastly, saliency maps were used to derive representations of the relevant spatio-temporal features that drove CNN decisions. MS-EEGNet was the lightest CNN compared with the tested SOA CNNs, despite its multiple timescales, and significantly outperformed the SOA algorithms. *Post-hoc* hyper-parameter analysis confirmed the benefits of the innovative aspects of MS-EEGNet. Furthermore, MS-EEGNet did benefit from transfer learning, especially using a low number of training examples, suggesting that the proposed approach could be used in BCIs to accurately decode the P300 event while reducing calibration times. Representations derived from the saliency maps matched the P300 spatio-temporal distribution, further validating the proposed decoding approach. This study, by specifically addressing the aspects of lightweight design, transfer learning, and interpretability, can contribute to advance the development of deep learning algorithms for P300-based BCIs.

## Introduction

The P300 response is an attention-dependent event-related potential (ERP) first reported in electroencephalographic (EEG) signals by Sutton et al. (1965). This wave is characterized by a positive deflection that peaks within the time window between 250 and 500 ms after stimulus onset, and it is mostly distributed on the scalp around the midline EEG electrodes (Fz, Cz, Pz), increasing its magnitude from the frontal to the parietal sites (Polich, 2007). The P300 can be evoked in an oddball paradigm (Farwell and Donchin, 1988), where an infrequent deviant stimulus immersed in a sequence of frequent standard stimuli is presented to the user while he/she is attending to it (e.g., by counting how many times a rare event occurs). Rare events induce the P300 response; this response can be used as a neural signal in EEG-based brain-computer interfaces (BCIs), enabling direct communication between the brain and surroundings without the involvement of peripheral nerves or muscles (Nicolas-Alonso and Gomez-Gil, 2012). One of the first P300-based BCIs was developed by Farwell and Donchin (1988) using a visual stimulation in the oddball paradigm. These systems could be especially beneficial for patients suffering from motor neuron disease (Rezeika et al., 2018) to provide alternative ways of communication. Furthermore, they may represent viable training tools for patients with attention deficits as recently reported in Amaral et al. (2018) where a P300-based BCI paradigm was tested in patients suffering from autism spectrum disorder (ASD) to improve their social attention.

Of course, a crucial aspect of a P300-based BCI is the decoding algorithm that translates brain signals into classes (e.g., P300 and non-P300 classes). Machine learning (ML) techniques have been recognized to be powerful tools in learning discriminative patterns from brain signals. In recent years, deep learning, a branch of ML originally proposed in computer vision (Guo et al., 2016; Ismail Fawaz et al., 2019), has been applied to decoding problems of physiological signals, such as electroencephalography, electromyography, electrocardiography, and electrooculography (Faust et al., 2018). At variance with more traditional ML approaches characterized by a separation between feature extraction, selection and classification stages (LeCun et al., 2015), deep learning techniques automatically learn features from raw or light pre-processed inputs to maximize between-class discriminability and finalize the decoding task in an end-to-end fashion.

Among deep learning techniques for classification, convolutional neural networks (CNNs) are widely used. These are specialized feed-forward neural networks involving the convolution operator to process data with a grid-like topology and are inspired by the hierarchical structure of the ventral stream of the visual system. Stacking neurons with a local receptive field on top of others creates receptive fields of individual neurons that increase in size in deeper layers of the CNN and increases the complexity of the features to which the neurons respond (Lindsay, 2020), realizing different levels of feature abstraction. This way, CNNs automatically learn hierarchically structured features from the input data, finalized to the classification. However, CNNs have some weaknesses: they introduce a large number of trainable parameters (consequently requiring a large number of training examples), they introduce many hyper-parameters (i.e., parameters that define the functional form of decoder), and learned features are difficult to be interpreted.

The field of EEG classification (and in particular P300 classification) has been widely exploiting the advantages of CNNs (Faust et al., 2018; Craik et al., 2019). At the same time, solutions to mitigate the weaknesses of these algorithms have been proposed within this field, as reported in the state-of-the-art (SOA) description below.

In CNN-based EEG classification, EEG signals can be arranged into a 2D representation with electrodes along a dimension and time steps along the other, and fed as input to the CNN that predicts the corresponding label. CNN designs for EEG classification include both shallow and deep neural networks, and solutions have been proposed either by performing spatial and temporal convolutions together (i.e., mixed spatio-temporal feature learning) or separately (i.e., unmixed spatio-temporal feature learning). Among the latter, several have been successfully applied to P300 classification (Cecotti and Graser, 2011; Manor and Geva, 2015; Lawhern et al., 2018; Liu et al., 2018; Shan et al., 2018; Farahat et al., 2019) and generally have been proved to outperform traditional ML approaches. Cecotti and Graser (2011) designed a CNN comprising two convolutional and two fully-connected layers to decode the P300 event. Remarkably, this was also the first attempt of CNN-based P300 decoding. Extensions of this architecture mainly focused on the increase of depth, and inclusion of batch normalization and dropout (Manor and Geva, 2015; Liu et al., 2018). Moreover, Farahat et al. (2019) proposed a dual-branched CNN (BranchedNet) that learns temporal features in two different timescales with parallel temporal convolutions, reporting an increase in performance with respect to a single-scale convolution. While these CNNs performed better than traditional ML techniques in P300 decoding, two aspects deserve attention: (i) they learn spatial features (i.e., spatial convolution, performed across electrodes) and then temporal features in the next layers (i.e., temporal convolution, performed across time samples); (ii) they do not address the challenge of reducing the number of trainable parameters. Regarding the first aspect, Shan et al. (2018) pointed out that these architectures may lose useful raw temporal information related to the P300 event since temporal features are learned from spatially filtered signals instead of from raw inputs. The authors proved that an architecture with the first layer performing a mixed spatio-temporal convolution (OCLNN) improved the decoding performance compared with the architecture proposed by Cecotti and Graser (2011) and other variants (Manor and Geva, 2015; Liu et al., 2018). Regarding the second aspect, recently, Lawhern et al. (2018) have designed a shallow CNN for EEG decoding, which is also applied to P300 detection (EEGNet). This design, besides performing temporal convolution in the first layer, uses separable and depthwise convolutions, i.e., convolutions specifically devoted to reducing the number of trainable parameters (Chollet, 2016).

Remarkably, recently, we have proposed a CNN (Borra et al., 2020a) based on the design of EEGNet that won the P300 decoding challenge issued by the International Federation of Medical and Biological Engineering (IFMBE) in 2019, where the dataset (BCIAUT-P300) was a large multi-participant and multi-session collection of data. The solution of the authors outperformed significantly a CNN derived from Manor and Geva (2015) with a spatial convolutional layer as the first layer, long short-term memories, and traditional ML approaches (Simões et al., 2020). These results further substantiate that CNNs, which include a temporal convolutional layer as the first layer, can represent advantageous solutions for P300 decoding compared with traditional approaches and other CNN designs.

Techniques have been proposed for interpreting and understanding what the CNN has learned (Montavon et al., 2018); in the field of EEG classification, they are fundamental to validate correct learning, checking that the learning system does not rely on artifactual sources but on neurophysiological features. These techniques explain the decoding decision taken by the CNN, i.e., features on which the CNN mainly relies to discriminate among classes. In this way, they represent tools to explore and analyze the underlying neurophysiology potentially characterizing new features (unknown so far) and gaining insights into neural correlates of the underlying phenomena. Montavon et al. (2018) provided a definition for *explanation of CNN decision*: “the collection of features of the interpretable domain, that have contributed for a given example to produce a decision (e.g., classification or regression).” Among the explanation techniques proposed in the computer vision domain (Montavon et al., 2018), saliency maps (Simonyan et al., 2013), simple representations reporting the gradient of a target class score with respect to each input pixel, have been recently transposed to P300 decoding (Farahat et al., 2019). Furthermore, other techniques were adopted to understand CNNs for P300 decoding, such as temporal and spatial kernel visualizations (Cecotti and Graser, 2011; Lawhern et al., 2018), and kernel ablation tests (Lawhern et al., 2018). In addition to these techniques, interpretable layers (where the learned features are directly interpretable without the need for *ad hoc* techniques) were recently applied to EEG decoding tasks (Zhao et al., 2019; Borra et al., 2020b,c).

Within this field of research, the aim of this study is to further contribute to the development of CNNs for EEG-based P300 decoding and to their analysis, with particular emphasis on the following aspects: keeping limited the number of trainable parameters (also referred to as model size) to realize lightweight CNNs suitable also for small datasets; assessing the effects of different learning strategies (including transfer learning) in view of the practical usage of these algorithms in BCIs; explaining the CNN decision i.e., the neurophysiological aspects that resulted in an optimal discriminability between classes. Specifically, the main contribution points are the following:

- The realization of a CNN named MS-EEGNet combining two designs previously proposed in the literature with unique characteristics but treated separately, with the aim of jointly exploiting their respective strengths (see section MS-EEGNet). On one hand, we adopted a branched architecture in order to extract features in two different timescales, since this may improve the performance of P300 decoding (as suggested by Farahat et al., 2019). On the other hand, the branched solution would tend to increase the number of convolutional layers (since convolutions are replicated along each branch) and consequently the number of trainable parameters. Therefore, we adopted solutions to keep limited the number of trainable parameters by limiting the overall number of convolutional layers (designing a shallow network) and at the same time implementing computationally efficient convolutions, such as depthwise and separable convolutions (as adopted by Lawhern et al., 2018). The latter are characterized by a reduced number of required multiplications, hence by a lower computational cost, and by a reduced number of trainable parameters compared with conventional convolutions (as those adopted by Farahat et al., 2019). In addition, learning compressed temporal representations in MS-EEGNet helped to further reduce the overall model size. In this way, we proposed a multi-scale lightweight design. The so obtained network was then thoroughly analyzed to evaluate its performance and potentialities in view of practical applications (see points below).
- Analysis of the main hyper-parameters of the architecture, evaluating variant designs to investigate the role of multi-scale temporal feature learning (see section Alternative Design Choices of MS-EEGNet: Changing Hyper-parameters in the MST Block).
- Application of MS-EEGNet to three different datasets, to evaluate the proposed approach on variable-sized datasets and on differently elicited P300 responses, comparing the performance with other SOA algorithms, including both CNNs and a traditional ML pipeline (see sections Data and Pre-processing and State-of-the-Art Algorithms).
- Training of MS-EEGNet with different strategies that include transfer learning. Transfer learning is of relevance as it could provide important benefits in practical BCI applications, alleviating the need for a large training set and reducing training times when using the CNN on a new user (see section Training).
- Application of an explanation technique based on saliency maps to derive the spatial and temporal features that drove MS-EEGNet decision (see section Explaining P300 Decision: Gradient-Based Representations).

## Materials and Methods

In this section, first, we introduce the problem of EEG decoding *via* CNNs. Then, we describe the proposed architecture in its baseline and variant versions, P300 datasets, re-implemented SOA algorithms, training strategies, and CNN explanation. Lastly, we illustrate the adopted statistical analyses.

Convolutional neural networks were developed in PyTorch (Paszke et al., 2017) and trained using a workstation equipped with an AMD Threadripper 1900X, NVIDIA TITAN V, and 32 GB of RAM. Codes of MS-EEGNet are available at https://github.com/ddavidebb/P300_decoding_MS-EEGNet.

### EEG Decoding *via* CNNs

Let us consider an EEG dataset collected from many participants and recording sessions. Each single participant- and session-specific dataset is composed of many trials collected by epoching the continuous EEG recording with respect to the onset of the stimulus (e.g., standard or deviant stimulus). Thus, each trial is associated with a specific class (e.g., non-P300 or P300 class), with a total of *N*_*c*_ classes. Indicating with *M*^(*s, r*)^ the total number of trials for the s-th subject and the r-th recording session, the corresponding dataset can be formalized as: $D^{\left(s,r\right)}=\left\{\left(X_{0}^{\left(s,r\right)},\;y_{0}^{\left(s,r\right)}\right),\;\ldots ,\left(X_{i}^{\left(s,r\right)},\;y_{i}^{\left(s,r\right)}\right),\ldots ,\left(X_{M^{\left(s,r\right)}-1}^{\left(s,r\right)},\;y_{M^{\left(s,r\right)}-1}^{\left(s,r\right)}\right)\right\}$. $X_{i}^{\left(s,r\right)}\in {\mathbb{R}}^{C\times T}$ represents the pre-processed EEG signals of the i-th trial (0 ≤ *i* ≤ *M*^(*s, r*)^−1), with *C* indicating the number of electrodes and *T* indicating the number of time steps. $y_{i}^{\left(s,r\right)}$ is the label associated with $X_{i}^{\left(s,r\right)}$, i.e., $y_{i}^{\left(s,r\right)}\in L=\left\{l_{0},\ldots ,l_{N_{c}-1}\right\}$. In the particular case of P300 decoding, i.e., discrimination between standard and deviant trials, *N*_*c*_ = 2 and $L\;=\;\left\{l_{0},l_{1}\right\}=\left\{“non-P300,“P300^{\prime \prime }\right\}$.

The objective decoding problem can be formalized as the optimization of a parametrized classifier *f* implemented by a CNN, $f\left(X_{i}^{\left(s,r\right)};\theta \right):{\mathbb{R}}^{C\times T}\to L$, with parameters θ, learning from a training set to assign the correct label to unseen EEG trials. Therefore, in the following, we refer to $X_{i}^{\left(s,r\right)}$ as the CNN input, represented as a 2D matrix of shape(*C, T*) with time steps along the width and electrodes along the height. Lastly, each dataset *D*^(*s, r*)^ was divided into a training set used to optimize the parameters contained in array θ, and a test set used to evaluate the algorithm on unseen data. Furthermore, a separate validation set needs to be extracted from the training set to define a stop criterion of the optimization. As described in section Data and Pre-processing, here, we used three datasets: dataset 1 was a large public dataset where each participant performed different recording sessions, while datasets 2 and 3 were two small private datasets where each participant performed a single recording session.

### The Proposed Convolutional Neural Network and Its Variants

#### MS-EEGNet

**Figure 1:**
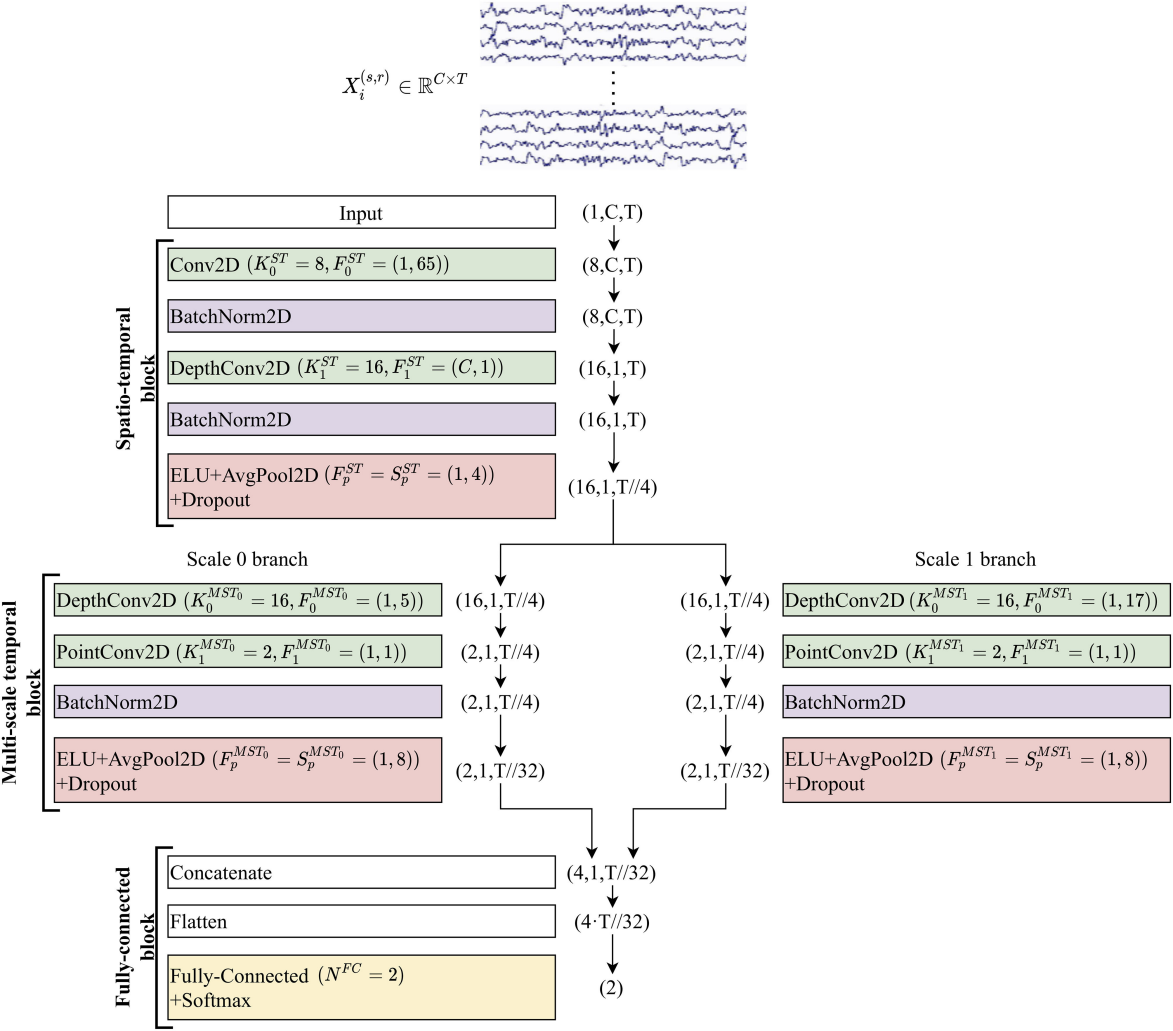
Structure of MS-EEGNet. Layers are represented by colored rectangles, reporting the layer name and main hyper-parameters. The tuple outside each rectangle represents the output shape of each layer. For all outputs except the last two (Flatten and Fully-connected + Softmax), the tuples are composed of three numbers representing the number of feature maps (channel dimension), number of spatial samples, and number of temporal samples within each map. The input layer provides an output of shape (1, *C, T*), as it just replicates the original input matrix with shape (*C, T*), providing a single feature map as output. The temporal dimension changed from *T* to *T*//32 along the entire CNN (where the symbol // indicates the floor division operator) due to average pooling operations. See sections EEG Decoding via CNNs and The Proposed Convolutional Neural Network and Its Variants for the meaning of symbols, and see Table 1 for further details.

The proposed shallow architecture was composed of three fundamental blocks, each consisting of many layers. A schematic representation of the CNN is reported in Figure 1. The spatio-temporal (ST) block extracted temporal and spatial features from the input EEG signals *via* temporal and spatial convolutional layers, respectively. Downstream, the multi-scale temporal (MST) block used lightweight parallel temporal convolutions to extract temporal patterns in different scales from the feature maps provided by the previous block. Lastly, multi-scale activations were provided to the fully-connected (FC) block that finalized the decoding task using a single fully-connected layer.

In all the layers except for the last two, the output was a collection of spatio-temporal feature maps and its shape can be described by a tuple of three integers, with the first integer indicating the number of feature maps, and the second and third integers representing the number of spatial and temporal samples within each map, respectively. In the following, to describe the CNN, we will refer to the hyper-parameters of the involved layers. Each convolutional layer is characterized by the number of convolutional kernels (*K*), kernel size (*F*), stride size (*S*), and padding size (*P*). In addition, depthwise convolution introduced also a depth multiplier (*D*) specifying the number of kernels to learn for each input feature map. Hyper-parameters will be denoted by a superscript and a subscript. The superscript indicates the specific block to which the layer belongs using acronyms “ST,” “MST_0_,” “MST_1_,” and “FC,” where the index in the MST block discriminates between the two scales (in general *MST*_*i*_, where 0 ≤ *i* ≤ *N*_*b*_−1 and *N*_*b*_ denotes the number of parallel branches). The subscript indicates to which convolutional layer inside the block the hyper-parameters refer (convolutional layers inside each block were labeled with an increasing index, starting from 0). Lastly, pooling layers were described by pool size (*F*_*p*_) and pool stride (*S*_*p*_), with the corresponding superscript. Both convolutions and poolings were 2D; therefore, *F*, *S*, *P*, *F*_*p*_, and *S*_*p*_ were tuples of two integers: the first referred to the spatial dimension, while the second referred to the temporal dimension. Lastly, the number of time samples changed across pooling operators and was denoted with *T*_*p*_. Regarding the single fully-connected layer included in the classification block, the number of neurons was denoted with *N*^*FC*^ and represented the number of classes to decode (*N*_*c*_).

MS-EEGNet was analyzed in a baseline version and in many variants by adopting a *post-hoc* hyper-parameter evaluation procedure on the main MST block hyper-parameters. The baseline version is described in the current section, where the structure and function of each block are presented, while the variants are described in section Alternative Design Choices of MS-EEGNet: Changing Hyper-parameters in the MST Block.

i. Spatio-temporal block. This was designed to learn temporal and spatial features separately. At first, a temporal convolutional layer was included, learning ${K_{0}}^{ST}=8$ temporal kernels with filter size ${F_{0}}^{ST}=\left(1,65\right)$, unitary stride and zero padding ${P_{0}}^{ST}=\left(0,32\right)\;$to preserve the number of input temporal samples. Then, the ${D_{1}}^{ST}=2$ spatial filters of size (*C*, 1) were learned for each temporal feature map in a spatial depthwise convolutional layer, with unitary stride and without zero padding (Lawhern et al., 2018; Borra et al., 2020a). Thus, a total number of ${K_{1}}^{ST}={K_{0}}^{ST}\cdot {D_{1}}^{ST}=16$ spatial filters were learned and constrained to have a norm upper bounded by *c* = 1 (kernel max-norm constraint) as in previous studies (Lawhern et al., 2018; Borra et al., 2020a; Vahid et al., 2020). The feature maps of this layer were not fully connected with the feature maps of the previous layer. This not only reduced the number of trainable parameters but also allowed more straightforward spatio-temporal feature learning. Indeed, each group of *D*_1_ spatial filters was related to a specific temporal filter (Lawhern et al., 2018) (i.e., to specific spectral information). Furthermore, the output activations of the temporal and spatial convolutional layers were normalized *via* batch normalization (Ioffe and Szegedy, 2015). Downstream the spatial depthwise convolution and its associated batch normalization, the neurons were activated *via* an exponential linear unit (ELU) non-linearity (Clevert et al., 2015), i.e., *f*(*x*) = *x, x*>0 and *f*(*x*) = α(exp(*x*)−1), *x* ≤ 0. We adopted this activation function, since it was proved to allow faster and more noise-robust learning than other non-linearities (Clevert et al., 2015) and to outperform other activation functions when using CNNs with EEG signals (Schirrmeister et al., 2017). The α hyper-parameter controls the saturation value for negative inputs, and α = 1 was set here. Then, an average pooling layer was introduced to reduce the size of the activations along the temporal dimension from *T* to ${T_{p}}^{ST}$, with a pool size of ${F_{p}}^{ST}=\left(1,4\right)$ and pool stride of ${S_{p}}^{ST}=\left(1,4\right)$, providing activations sampled in 1/4 of the sampling frequency of the signals (32 Hz when using signals extracted from dataset 1 and approximately 31.3 Hz from datasets 2 and 3). Lastly, a dropout layer (Srivastava et al., 2014) (with a different dropout rate *p* depending on the training strategy adopted, see section Training) was added.
ii. Multi-scale temporal block. This block was designed to learn how to summarize along the temporal dimension the feature maps provided by the ST block. Differently from EEGNet where features in a single timescale were learned at this stage, here the features were learned in *N*_*b*_ different timescales, inspired by the design of the Inception modules (Szegedy et al., 2015). In the baseline MS-EEGNet *N*_*b*_ = 2, thus, two different sets of short and large kernels were separately learned in the two parallel branches. This was accomplished *via* two parallel temporal depthwise convolutional layers with a unitary depth multiplier, i.e., ${D_{0}}^{MST_{0}}={D_{0}}^{MST_{1}}={D_{0}}^{MST}=1$ and ${K_{0}}^{MST_{0}}={K_{0}}^{MST_{1}}={K_{0}}^{MST}={K_{1}}^{ST}\cdot {D_{0}}^{MST}$, and with different kernel sizes in the two branches extracting a summary of roughly 150 ms [${F_{0}}^{MST_{0}}=\left(1,5\right)$] and 500 ms [${F_{0}}^{MST_{1}}=\left(1,17\right)$], for each input feature map. That is, each output feature map was a sort of weighted moving average of the input feature map using moving windows of two different lengths, ~150 and 500 ms (referred to as scales). The large kernel size was chosen to match the temporal kernel size used in the single-scale branch of EEGNet (Borra et al., 2020a). The small kernel size was chosen so that the ratio between the small and large kernels was approximately the same as that in BranchedNet ($r^{MST}=\frac{1}{4}$), keeping odd kernel size (i.e., 500 ms/4 = 125 ms = four samples at 32 Hz, approximated to five samples to have an odd integer). The small and large temporal filters should be able to learn high and low-frequency patterns from the input, respectively (Supratak et al., 2017). Here, unitary stride and zero-padding of ${P_{0}}^{MST_{0}}=\left(0,2\right)$ and ${P_{0}}^{MST_{1}}=\left(0,8\right)$ were adopted, preserving the number of the input temporal samples. After each depthwise convolutional layer, a pointwise convolutional layer [${F_{1}}^{MST_{0}}={F_{1}}^{MST_{1}}={F_{1}}^{MST}=\left(1,\;1\right)$] was added to learn how to optimally combine the feature maps in a specific timescale with unitary stride and without zero-padding. At variance with BranchedNet (Farahat et al., 2019) where convolutions were not designed to keep limited the number of trainable parameters, the proposed multi-scale temporal block was designed using separable convolutions (i.e. depthwise convolution followed by pointwise convolution) with the specific aim of reducing the training parameters. In this same perspective, the number of output feature maps was set as low as ${K_{1}}^{MST_{0}}={K_{1}}^{MST_{1}}={K_{1}}^{MST}=2$ in each branch, learning a compressed representation of the input feature maps (i.e., the 16 input feature maps provided by the depthwise convolutional layer were recombined into only two different feature maps, for each branch). Then, for each branch, the output activations of the pointwise convolutional layer were normalized *via* batch normalization (Ioffe and Szegedy, 2015) and activated with an ELU non-linearity (α = 1). Finally, an average pooling layer was introduced with a pool size of ${{{F_{p}}^{MST_{0}}=F}_{p}}^{MST_{1}}={F_{p}}^{MST}=\left(1,\;8\right)$ and pool stride of ${S_{p}}^{MST_{0}}={S_{p}}^{MST_{1}}={S_{p}}^{MST}=\left(1,\;8\right)$ to reduce the temporal dimension from ${T_{p}}^{ST}$ to ${T_{p}}^{MST}$, followed by a dropout layer (Srivastava et al., 2014) (with different dropout rate *p* depending on the training strategy adopted, see section Training).
iii. Fully-connected block. This block was devoted to produce output probabilities from the feature maps provided by the multi-scale temporal block. The input feature maps were concatenated together along the feature map dimension and unrolled along a single dimension *via* a flatten layer. Then, this multi-scale feature vector was given as input to a fully-connected layer with ${N^{FC}=N}_{c}=2$ neurons (associated with the P300 and non-P300 classes). These two outputs were transformed via a Softmax activation function to obtain conditional probabilities $p\left(l_{k}|X_{i}^{\left(s\right)}\right)$, *k* = 0, 1.

A more detailed description of the structural hyper-parameters and of the number of trainable parameters of the baseline version of MS-EEGNet can be found in Table 1. The overall number of trainable parameters (or model size) and the training time (or computational time) of the baseline MS-EEGNet are reported in Table 2. Note that in this table, these variables are reported also for the variant designs of MS-EEGNet (see section Alternative Design Choices of MS-EEGNet: Changing Hyper-parameters in the MST Block) and for the examined SOACNNs (see section State-of-the-Art Algorithms).

**Table 1:** Architecture details of MS-EEGNet.

**Block**	**Layer name**	**Hyper-parameters**	**Number of trainable parameters**
	Input	*K*_0_ = 1	0
ST	Conv2D	${K_{0}}^{ST}=8$, ${F_{0}}^{ST}=\left(1,65\right)$, ${P_{0}}^{ST}=\left(0,32\right)$	${F_{0}}^{ST}\left[0\right]{{\cdot F}_{0}}^{ST}\left[1\right]\cdot {K_{0}}^{ST}\cdot K_{0}$
	BatchNorm2D	*m* = 0.99	$2\cdot {K_{0}}^{ST}$
	Depthwise-Conv2D	${D_{1}}^{ST}=2$, ${K_{1}}^{ST}={K_{0}}^{ST}\cdot {D_{1}}^{ST}$, ${F_{1}}^{ST}=\left(C,1\right)$, kernel max norm=1	${F_{1}}^{ST}\left[0\right]{{\cdot F}_{1}}^{ST}\left[1\right]\cdot {K_{0}}^{ST}\cdot {D_{1}}^{ST}$
	BatchNorm2D	*m* = 0.99	$2\cdot {K_{1}}^{ST}$
	ELU	α = 1	0
	AvgPool2D	${F_{p}}^{ST}={S_{p}}^{ST}=\left(1,4\right)$	0
	Dropout	*p* = 0.25 or *p* = 0.5	0
MST scale 0	Depthwise-Conv2D	${D_{0}}^{MST_{0}}=1$, ${K_{0}}^{MST_{0}}={K_{1}}^{ST}\cdot {D_{0}}^{MST_{0}}$, ${F_{0}}^{MST_{0}}=\left(1,5\right)$, ${P_{0}}^{MST_{0}}=\left(0,2\right)$	${F_{0}}^{MST_{0}}\left[0\right]\cdot {F_{0}}^{MST_{0}}\left[1\right]\cdot {K_{1}}^{ST}\cdot {D_{0}}^{MST_{0}}$
	Pointwise-Conv2D	${K_{1}}^{MST_{0}}=2$, ${F_{1}}^{MST_{0}}=\left(1,1\right)$	${F_{1}}^{MST_{0}}\left[0\right]\cdot {F_{1}}^{MST_{0}}\left[1\right]\cdot {K_{1}}^{MST_{0}}{{\cdot K}_{0}}^{MST_{0}}$
	BatchNorm2D	*m* = 0.99	$2\cdot {K_{1}}^{MST_{0}}$
	ELU	α = 1	0
	AvgPool2D	${F_{p}}^{MST_{0}}={S_{p}}^{MST_{0}}=\left(1,8\right)$	0
	Dropout	*p* = 0.25 or *p* = 0.5	0
MST scale1	Depthwise-Conv2D	${D_{0}}^{MST_{1}}=1$, ${K_{0}}^{MST_{1}}={K_{1}}^{ST}\cdot {D_{0}}^{MST_{1}}$, ${F_{0}}^{MST_{1}}=\left(1,17\right)$, ${P_{0}}^{MST_{1}}=\left(0,8\right)$	${F_{0}}^{MST_{1}}\left[0\right]\cdot {F_{0}}^{MST_{1}}\left[1\right]\cdot {K_{1}}^{ST}\cdot {D_{0}}^{MST_{1}}$
	Pointwise-Conv2D	${K_{1}}^{MST_{1}}=2$, ${F_{1}}^{MST_{1}}=\left(1,1\right)$	${F_{1}}^{MST_{1}}\left[0\right]\cdot {F_{1}}^{MST_{1}}\left[1\right]\cdot {K_{1}}^{MST_{1}}{{\cdot K}_{0}}^{MST_{1}}$
	BatchNorm2D	*m* = 0.99	$2\cdot {K_{1}}^{MST_{1}}$
	ELU	α = 1	0
	AvgPool2D	${F_{p}}^{MST_{1}}{{=S}_{p}}^{MST_{1}}=\left(1,8\right)$	0
	Dropout	*p* = 0.25 or *p* = 0.5	0
FC	Concatenate Flatten		0
	Fully-Connected	*N*^*FC*^ = 2	$N^{FC}\cdot \left({T_{p}}^{MST_{0}}{{{{\cdot K}_{1}}^{MST_{0}}+T}_{p}}^{MST_{1}}{{\cdot K}_{1}}^{MST_{1}}+1\right)$
	Softmax		0

*Each layer is provided with its name, main hyper-parameters and the number of trainable parameters. See sections EEG Decoding via CNNs and The Proposed Convolutional Neural Network and Its Variants for the meaning of symbols. The total number of trainable parameters was 1,154 when using signals from dataset 1 and 1,210 when using signals from datasets 2 and 3. In all layers, unless otherwise noted stride (S) and padding (P) were set to (1, 1) and (0, 0), respectively*.

**Table 2:** Number of trainable parameters, also denoted as model size in the text, and training time (referred to the WS strategy), also denoted as computational time, of the baseline MS-EEGNet, MS-EEGNet variants, and SOA CNNs when using signals from dataset 1 and datasets 2–3.

**Algorithm**	**Trainable parameters**		**Training time**	
	**(dataset 1/datasets 2–3)**		**(dataset 1/datasets 2–3)**	
	**Value**	**Δ**	**Value**	**Δ**
		**(%)**	**(ms/epoch)**	**(%)**
*Baseline MS-EEGNet*	1,154/1,210	–	220/45.5	–
***MS-EEGNet variants***				
*N*_*b*_ = 1(*large*)	1,022/1,082	−11.4/−10.6	195/38.1	−11.4/−16.3
*N*_*b*_ = 1(*short*)	830/890	−28.1/−26.5	172/38.4	−21.8/−15.6
*N*_*b*_ = 3	1,350/1,402	17.0/15.9	282/50.8	28.2/11.6
${F_{0}}^{MST_{0}}=\left(1,9\right)$	1,218/1,274	5.5/5.3	221/46.3	0.5/1.8
${K_{1}}^{MST}=1$	1,102/1,162	−4.5/−4.0	224/45.0	1.8/−1.1
${K_{1}}^{MST}=8$	1,466/1,498	27.0/23.8	287/46.1	30.5/1.3
${K_{1}}^{MST}=16$	1,882/1,882	63.1/55.5	240/45.0	9.0/−1.1
deepMST	1,202/1,258	4.2/4.0	295/47.0	34.1/3.3
***SOA CNNs***				
*EEGNet*	1,386/1,418	20.1/17.2	186/40.5	−15.5/−11.0
*BranchedNet*	5,418/7,954	369/557	250/50.3	13.6/10.5
*OCLNN*	1,650/1,874	43.0/54.9	96.2/22.9	−56.3/−49.7

*These values were reported for deep learning-based decoders to provide a more complete comparison between the proposed CNN and SOA CNNs. For each CNN, between dataset 1 and datasets 2–3, the different number of parameters resulted from the different number of EEG channels (C = 8 for dataset 1 and C = 12 for datasets 2–3, see section Data and Pre-processing) and time samples considered (T = 140 for dataset 1 and T = 113 for datasets 2–3, see section Data and Pre-processing), while the different training time resulted from the different number of training examples (1,280 trials and 240 trials for each participant and each session, respectively, for dataset 1 and datasets 2–3, see section Data and Pre-processing). In addition, the percentage difference (Δ) of trainable parameters and training time between SOA CNNs or MS-EEGNet variants (“other” condition) and the baseline MS-EEGNet (“baseline” condition) is reported, i.e., 100·(value_other_−value_baseline_)/value_baseline_*.

#### Alternative Design Choices of MS-EEGNet: Changing Hyper-Parameters in the MST Block

In addition to the baseline MS-EEGNet described previously, we evaluated other alternative designs to better investigate the behavior of the proposed MST block by modifying some hyper-parameters (HPs) one at a time. In the following, the alternative designs are described and indicated *via* the modified HP: *HP*_*variant*_ vs. *HP*_*baseline*_.

- *N*_*b*_ = {1, 3} vs. *N*_*b*_ = 2: use of one or three branches. In this *post-hoc* analysis, we studied whether the proposed dual-scale temporal feature learning was beneficial compared with the traditional single-scale learning (*N*_*b*_ = 1) and which scale was able to learn more relevant class-discriminative temporal features. To this aim, MS-EEGNet was modified either by removing the short scale (scale 0), leaving only the large-scale branch [*N*_*b*_ = 1(*large*)] or the large scale (scale 1) leaving only the short-scale branch [*N*_*b*_ = 1(*short*)]. It is worth noticing that single-scale variant design *N*_*b*_ = 1 (large) did not correspond to the EEGNet adaptation used in Borra et al. (2020a), since here we adopted compressed representations in separable convolutional layers. In addition, we studied whether a third timescale (*N*_*b*_ = 3) could be useful by modifying MS-EEGNet by the inclusion of an additional timescale between the ones of the baseline version: and this variant learned summaries of about 125, 250, and 500 ms, corresponding to kernel sizes in the MST block of ${F_{0}}^{MST_{0}}=\left(1,5\right)$, ${F_{0}}^{MST_{1}}=\left(1,9\right)$, ${\text{and }F_{0}}^{MST_{2}}=\left(1,17\right)$, respectively.
- ${F_{0}}^{MST_{0}}=\left(1,9\right)$ vs. ${F_{0}}^{MST_{0}}=\left(1,5\right)$: enlarging the kernel size in the short-scale branch (scale 0 in Table 1). This was performed to evaluate the effect of a different ratio between the short- and large-scales of the MST block compared with the one adopted in the baseline MS-EEGNet. Specifically, $r^{MST}=\frac{1}{2}$ vs. $r^{MST}=\frac{1}{4}$ leading to 500 ms/2 = 250 ms = eight samples at 32 Hz, approximated to nine samples to have odd integer.
- ${K_{1}}^{MST}=\left\{1,8,16\right\}$ vs. ${K_{1}}^{MST}=2$: different number of feature maps in the pointwise convolutions. In particular, ${K_{1}}^{MST}$ was set to 1 in each branch in order to analyze whether the learning of a single recombination of the input feature maps was enough to provide an accurate decoding performance. In addition, ${K_{1}}^{MST}$ was set to 8 in each branch in order to analyze another compressed representation, while maintaining the total number of feature maps across the two different timescales unchanged as in the MST input (i.e., eight feature maps in each branch, resulting in 16 feature maps across the two scales, as in the input of the MST block). Lastly, ${K_{1}}^{MST}$ was set to 16 in each branch, corresponding to a condition where no compressed representation was learned in either branch.
- Deep MST vs. MST: increasing the depth of the MST block. This was performed to evaluate the effect on the performance of an increased depth in the MST block (and thus, learning more non-linear dependencies) while maintaining the same overall receptive field of the neurons in the temporal domain. In each branch, we added another depthwise convolutional layer after the first one. However, in order to maintain the same receptive field as when using a single depthwise convolutional layer in the baseline MST block, the kernel size of each depthwise convolutional layer was halved with respect to the baseline values, i.e., ${F_{0}}^{MST_{0}}={F_{1}}^{MST_{0}}=\left(1,3\right)$ and ${F_{0}}^{MST_{1}}={F_{1}}^{MST_{1}}=\left(1,9\right)$. After the second depthwise convolutional layer, the pointwise convolutional layer was added [${F_{2}}^{MST_{0}}={F_{2}}^{MST_{1}}={F_{2}}^{MST}=\left(1,\;1\right)$], and the rest of the block was maintained unchanged as in the baseline version.

Overall, eight variants were designed by changing a specific hyper-parameter value of MS-EEGNet while keeping all the other hyper-parameters as in the baseline MS-EEGNet. These alternative designs were trained with a within-participant and within-session strategy (as it is the most common strategy adopted in the literature) and compared with MS-EEGNet trained with the same strategy. Lastly, the number of trainable parameters and training time are reported in Table 2 for each variant design.

### Data and Pre-processing

#### Dataset 1

The first dataset is BCIAUT-P300, a public benchmark dataset released for the IFMBE 2019 scientific challenge (available at https://www.kaggle.com/disbeat/bciaut-p300) (Simões et al., 2020) consisting of a larger number of examples than other public benchmarks (Blankertz et al., 2004, 2006) or private (Lawhern et al., 2018; Farahat et al., 2019; Solon et al., 2019) datasets. Signals were recorded from 15 participants (all males, age of 22 ± 5 years, mean ± standard deviation) with ASD during seven recording sessions (for a total of 4 months) while testing a P300-based BCI (Amaral et al., 2018). The paradigm consisted of the participants paying attention to one of eight objects randomly flashing in a virtual scene, with P300 stimuli corresponding to the flashing of the attended object (this was repeated several times for each different attended object). For each participant and recording session, 1,600 trials were recorded during the calibration stage (training set), and 2,838 trials were recorded during the online stage (test set), on average.

Signals were recorded at 250 Hz from eight electrodes: C3, Cz, C4, CPz, P3, Pz, P4, and POz. The reference was placed at the right ear and the ground at AFz. These signals were acquired notch filtered at 50 Hz and then pass-band filtered between 2 and 30 Hz (Simões et al., 2020). EEG signals were pre-processed as in previous studies (Amaral et al., 2017; Borra et al., 2020a). In particular, epochs were selected from −100 to 1,000 ms relative to the event stimulus, and signals were downsampled to 128 Hz to reduce the number of time steps to be processed in the CNN. Architectures were trained as described in section Training using the training set of the competition for each session, while the test set was used to test the algorithms. From each participant- and session-specific training set, a validation set of 20% of the total training set was extracted (corresponding to 320 trials) to perform early stopping, while the remaining percentage of the total training set (corresponding to 1,280 trials) was used to optimize the architectures.

#### Datasets 2 and 3

The second dataset was collected from seven participants (all males, age 25 ± 8 years) recorded in an auditory oddball study during a single recording session, and the third dataset was collected from seven participants (5 males, age 22 ± 0.4 years, different from dataset 2 participants) recorded in a visual oddball study during a single recording session. All the participants were healthy volunteers not reporting psychological or hearing disorders. Both experiments were approved by the Bioethics Committee of the University of Bologna (file number 29146, year 2019) and were conducted in a controlled laboratory environment.

The auditory oddball paradigm consisted of 400 tones presented to the participants through a speaker, with the standard and deviant stimuli differing by the frequency of tones (500 and 1,000 Hz, respectively). The visual oddball paradigm consisted of 400 stimuli presented to the participants through a bicolor LED with the standard and deviant stimuli differing by the LED color (blue and red, respectively). In both paradigms, each stimulus was reproduced for 56 ms followed by a pause of 944 ms (inter-stimuli interval); thus, each trial lasted 1 s. This paradigm was similar to the one adopted by Justen and Herbert (2018). Furthermore, in each paradigm, a total number of 325 standard and 75 deviant stimuli were presented to participants in a randomized order. Thus, for each participant, a total number of 400 trials were available, with a class imbalance ratio of 75:325 for the P300 and non-P300 classes. While listening to the tones or while looking at the LED, the participants were seated in a comfortable chair in front of a button with their eyes opened, and they were instructed to respond to the deviant stimuli by pressing a button with their right index finger as quickly as possible, minimizing other movements.

Signals of both datasets 2 and 3 were recorded at 125 Hz using a portable EEG recording system (OpenBCI system, using Cyton and Daisy Biosensing boards) from 12 electrodes: C3, Cz, C4, CP5, CP1, CP2, CP6, P3, Pz, P4, PO3, and PO4. The reference was placed at the right earlobe and the ground at the left earlobe. The same pre-processing was adopted for datasets 2 and 3. In particular, signals were band-pass filtered between 2 and 30 Hz with a zero-phase second-order filter, and epochs were extracted from −100 to 800 ms relative to the stimulus onset. For datasets 2 and 3, the architectures were trained as described in section Training using a 4-fold cross-validation scheme. Therefore, in each fold, each participant-specific dataset was divided into a training (75%) and a test (25%) set, corresponding to 300 and 100 trials, respectively. Lastly, a validation set of 20% of the training set (corresponding to 60 trials) was extracted to perform early stopping, while the remaining percentage (corresponding to 240 trials) was used to optimize the architectures.

As described in section EEG Decoding via CNNs, $X_{i}^{\left(s,r\right)}\in {\mathbb{R}}^{C\times T}$ represented the CNN input. From the previous dataset descriptions, *C* = 8 for dataset 1 and *C* = 12 for datasets 2 and 3, while *T* = 140 for dataset 1 and *T* = 113 for datasets 2 and 3.

### State-of-the-Art Algorithms

The proposed baseline architecture was compared with other SOA algorithms, such as the winning algorithm of the IFMBE 2019 challenge based on EEGNet (Borra et al., 2020a), BranchedNet (Farahat et al., 2019), and OCLNN (Shan et al., 2018). The first was a single-branched CNN performing the temporal convolution in the first layer. The second one, was a dual-branched CNN exploiting parallel temporal convolutions but at variance with the architecture proposed here, performed spatial convolution in the first layer and did not use optimized convolutions aimed to keep limited the number of trainable parameters, resulting in a less parsimonious multi-scale CNN. OCLNN was a CNN performing a mixed spatio-temporal convolution in the first layer without using optimized convolutions. To allow for a more complete comparison between MS-EEGNet and other deep learning-based decoders, the number of trainable parameters and training time of SOA CNNs are summarized in Table 2.

In addition to these SOA CNNs, we re-implemented xDAWN+RG, an ML pipeline for P300 decoding. In particular, this solution included a combination of xDAWN spatial filtering (Rivet et al., 2009; Barachant and Congedo, 2014), Riemannian Geometry (Barachant et al., 2012), *L*_1_ feature regularization, and classification based on an Elastic Net regression.

Details about SOA CNNs and xDAWN+RG can be found in sections 1 and 2 in Supplementary Materials.

### Training

MS-EEGNet was trained with different training strategies.

- Within-participant and within-session training (WS). For each participant and session, EEG signals (see section Data and Pre-processing) were used to train, validate, and test a participant-specific and session-specific CNN. In addition, we also trained CNNs using only a fraction of the participant- and session-specific training set, simulating practical cases of reduced numbers of available calibration trials, and investigated how the performance changed; this is an important issue from the perspective of limiting the calibration time in practical applications. Reduced training sets were defined by extracting 15, 30, 45, and 60% of the total training set in the corresponding session (corresponding to 192, 384, 576, and 768 training trials for dataset 1, and 48, 96, 144, and 192 trials for datasets 2 and 3, maintaining the class imbalance characterizing each dataset. For each architecture, 105 (15 participants ^*^ 7 sessions per participant) CNNs were trained for dataset 1, while seven (7 participants ^*^ 1 session per participant) CNNs were trained for datasets 2 and 3. The WS strategy (with 100% of training trials) was adopted also with SOA algorithms to perform *post-hoc* hyper-parameter evaluation.
- Within-participant and cross-session training (CS). This training strategy was adopted only for the dataset 1 because of its multi-session dimension and used in the winning solution of the authors in the IFMBE 2019 challenge (Borra et al., 2020a) using the same dataset. For each participant, an overall training set and an overall validation set were obtained by considering all the session-specific training and validation sets belonging to that particular participant. Then, these overall sets were used to train and validate a participant-specific CNN incorporating inter-session variability. It is worth noticing that this participant-specific CNN was then tested separately over each session-specific test set (relative to that participant) for consistency with the test procedure adopted in i). For each architecture, 15 CNNs were trained for dataset 1. This strategy was adopted also with SOA algorithms.
- Leave-one-subject-out training (LOSO). The EEG signals of one participant (i-th participant) were held back, and the training and validation sets were obtained by collecting EEG signals from all the session-specific training and validation sets of the remaining participants (j-th participants ∀*j, j*≠*i*). Thus, for each held back participant (∀*i*) an architecture was trained and validated with signals extracted from 14 participants for dataset 1 and from six participants for datasets 2 and 3. The so obtained network was then tested separately over each session-specific test set of the held back participant, consistently with the testing procedure in (i) and (ii). The residual signals of the held back participant not used in the testing procedure remained unused (i.e., 0% of the dataset of the held back participant was used to train and validate the model); that is, LOSO models did not learn from the examples of the held back participant. This training strategy led to a CNN incorporating inter-participant and (in case of dataset 1) inter-session variabilities. For each architecture, 15 CNNs were trained for dataset 1, while seven CNNs were trained for datasets 2 and 3. This strategy was adopted also with SOA algorithms. Lastly, to design LOSO models incorporating the knowledge from a variable number of participants, we additionally performed trainings extracting signals from a random subset of participants, i.e., using 10, six, and two participants for dataset 1, and using four and two participants for datasets 2 and 3. Thus, the performed LOSO strategy was named “LOSO-M,” where M is the number of participants used (*M* = {14, 10, 6, 2} when using signals from dataset 1, while *M* = {6, 4, 2} for datasets 2 and 3). It is worth noticing that the LOSO-14 strategy for dataset 1 and LOSO-6 strategy for datasets 2 and 3 corresponded to the conventional LOSO strategies for these datasets.
- Transfer learning (TL) on single sessions (WS). As in the WS strategy (point i), for each participant and session, EEG signals (see section Data and Pre-processing) were used to train, validate, and test a participant- and session-specific CNN. Differently from the WS strategy where the trainable parameters were initialized randomly, in the TL-WS strategy, the parameters were initialized from the ones obtained with LOSO trainings when the specific participant of interest was held back. Therefore, the knowledge learned in the LOSO strategy (using training examples sampled from many participants except the held back participant) was transferred to the held back participant. Then, a fraction of the session-specific training set of the held back participant was used as training set, using the same percentages as in the WS strategy (point i). In this way, we compared the performance of the WS and TL-WS strategies to investigate if and to what extent the TL-WS strategy outperformed the WS strategy with a reduced number of calibration trials. For each architecture, 105 CNNs were trained for dataset 1, while seven CNNs were trained for datasets 2 and 3.

The transfer learning strategy reflects a practical situation in which a new user approaches the BCI system in a new session, and a calibration phase, as short as possible, is needed to obtain an accurate participant-specific decoder. Therefore, a pre-trained model that incorporates both inter-participant and inter-session variabilities as obtained with the LOSO strategy could be a better initialization point with respect to the random one (as used in the WS training strategy), leading to performance improvement especially when using only a small number of training examples of a new user in a new recording session.

The adopted training strategies had a different definition of the training set. However, in all cases, CNNs were tested on the same participant-specific and session-specific test sets, allowing a fair comparison across different training strategies. In this study, the adopted metric to quantify the performance for the P300 decoding task at the trial level was the area under the ROC curve (AUC), as done previously (Lawhern et al., 2018), and was computed on each participant- and session-specific test set.

EEG signals of the training, validation, and test sets were standardized by computing the mean and variance on the training set. Regarding the TL-WS strategy, the first and second moments were computed on the training set used to train the pre-trained models. Except for the TL-WS strategy in which the trainable parameters were initialized from the pre-trained models, in the other training approaches, the weights were randomly initialized by adopting a Xavier uniform initialization scheme (Glorot and Bengio, 2010), and biases were initialized to zero.

The optimization was performed by minimizing the negative log likelihood or, equivalently, the cross-entropy between the empirical probability distribution defined by the training labels and the probability distribution defined by the model. Adaptive moment estimation (Adam) (Kingma and Ba, 2014) was used as an optimizer with β_1_ = 0.9, β_2_ = 0.999 for computing the running averages of the gradient and its square, and ε = 10^−8^ to improve numerical stability. The learning rate was set to *lr* = 10^−3^for the WS, CS, and LOSO strategies, while for the TL-WS strategy the optimizer state was the same as the one of the pre-trained models. To address class imbalance, a single mini-batch of data was composed by a proportion of 50–50% of the two classes, randomly selecting the trials within the dataset as done in Borra et al. (2020a). The mini-batch size and the maximum number of epochs were set to 64 and 500, respectively, and early stopping was performed by interrupting the optimization when validation loss did not decrease for 50 consecutive epochs.

In addition to early stopping, which acts as a regularizer, other regularizer mechanisms were integrated into MS-EEGNet as mentioned in section MS-EEGNet, comprising batch normalization (Ioffe and Szegedy, 2015) with a momentum term of *m* = 0.99 and ε = 1*e*−3 for numerical stability, dropout (Srivastava et al., 2014) with a dropout probability of 0.5 for WS and TL-WS trainings and 0.25 for CS and LOSO trainings, and kernel max-norm constraint.

### Explaining P300 Decision: Gradient-Based Representations

The MS-EEGNet decision was explained using the saliency maps and *post-hoc* (i.e., obtained once the CNN training has ended) gradient-based representations proposed by Simonyan et al. (2013) to quantify the importance of neurons belonging to a target layer of interest (commonly the input layer) for a specific class. These representations are commonly used to explain CNN decisions when decoding EEG (Farahat et al., 2019; Borra et al., 2020c; Vahid et al., 2020) and offer the advantage of requiring the sole computation of backpropagation. Of course, other more advanced techniques, such as layer-wise relevance propagation (LRP), can represent a valid alternative but they introduce many factors that affect representations, such as the propagation rule (e.g., αβ rule) and propagation parameters (e.g., α and β) (Montavon et al., 2018), whose setting would require preliminary deep investigations. Hence, we preferred to adopt the saliency maps. Here, these were computed by backpropagating the gradient of the P300 class score (i.e., the output related to the P300 neuron, immediately before Softmax activation) back to the input layer (i.e., the neurons corresponding to the input spatio-temporal samples), when P300 trials belonging to the test set were fed as input to the CNN. Thus, each resulting saliency map was a spatio-temporal representation associated with a test trial, quantifying how much each spatio-temporal input sample affects the P300 class score, i.e., how much the P300 class score changes with respect to a small change in the input EEG signals. For each dataset, these representations were computed using MS-EEGNet trained with the LOSO strategy, as this strategy was more likely to enhance input samples relevant to the decoding task compared with WS/CS trainings (Farahat et al., 2019). Indeed, during LOSO trainings, the models were fed with signals recorded from multiple participants and multiple recording sessions. Therefore, the neural networks were more prone to learn optimal inter-participant and inter-session features to generalize properly. Conversely, during WS/CS trainings, the neural networks were more prone to learn optimal session-specific/participant-specific features. Thus, representations associated with the LOSO models were more likely to visualize general task-relevant spatio-temporal features, while those related to the WS/CS models were more likely to include also session-specific/participant-specific and task-irrelevant features.

The saliency maps were computed for each deviant trial (containing the P300 response) belonging to each participant- and session-specific test set. Then, these maps were averaged across trials and folds (only for datasets 2 and 3), obtaining an average participant-specific and session-specific representation, named *spatio-temporal representation*. Then, by averaging spatio-temporal representations across sessions (seven sessions for dataset 1 and a session for datasets 2 and 3), a participant-specific representation was computed normalized between [−1, 1], and finally averaged across the participants, resulting in a *grand average (GA) spatio-temporal representation*. This representation could be useful to study similarities between the temporal course of gradients related to more relevant electrodes and the grand average ERPs of those specific electrodes. Additionally, the absolute value of each saliency map was also computed, and the absolute saliency maps were then averaged across trials, folds (only for datasets 2 and 3), and either the spatial or the temporal dimension to obtain an *absolute temporal or spatial representation*, respectively, for each participant and session. Then, by averaging the absolute temporal/spatial representation across sessions, a participant-specific representation was computed, normalized between [0, 1], and finally averaged across the participants, resulting in a *GA absolute temporal/spatial representation*. These absolute representations allowed the evaluation of more class-discriminative time samples and electrodes for the P300 class.

### Statistics

Before performing the statistical analyses, AUCs were computed for each participant- and session-specific test set and then averaged across sessions (seven sessions for dataset 1 and 1 session for datasets 2 and 3), in order to compare the performance metric at the level of participant. The following statistical comparisons were performed on the performance metric.

- Pairwise comparisons between MS-EEGNet and the SOA algorithms (EEGNet, BranchedNet, OCLNN, xDAWN+RG) trained with the WS, CS, and LOSO strategies. AUCs were compared between the contrasted conditions separately for each dataset.
- Pairwise comparisons between the baseline MS-EEGNet and each of its variants, trained with the WS strategy. The AUCs were merged together across different datasets and compared between the contrasted conditions using CNNs trained with the WS strategy; a similar procedure was adopted in Schirrmeister et al. (2017) and Borra et al. (2020c) in order to evaluate the overall effect of the hyper-parameters of interest with the *post-hoc* evaluation.
- Pairwise comparisons between MS-EEGNet trained with the WS and TL-WS strategies, for each percentage of training examples of the new user and for each number of participants (*M*) from whom the knowledge was transferred to the new user (see section Training-iv). This test was performed in order to evaluate the effect of the TL-WS strategy on the performance as a function of the percentage of training examples and *M*. In these pairwise comparisons, the AUCs were compared between the contrasted conditions separately for each dataset.

The statistical analysis performed was the same as that used in Schirrmeister et al. (2017) and Borra et al. (2020c). In particular, Wilcoxon signed-rank tests were used to check for statistically significant differences between the contrasted conditions. To correct for multiple tests, a false discovery rate correction at α = 0.05 using the Benjamini–Hochberg procedure (Benjamini and Hochberg, 1995) was applied.

## Results

### Performance

#### MS-EEGNet and State-of-the-Art Algorithms

Table 3 reports the AUCs at the participant level (mean ± standard error of the mean, SEM) obtained with MS-EEGNet and with SOA algorithms using WS, CS, and LOSO strategies, together with the results of the performed statistical tests.

**Table 3:** AUC (% mean ± SEM) obtained with MS-EEGNet and the re-implemented SOA algorithms adopting the WS, CS, and LOSO strategies.

**Algorithm**	**Dataset 1**			**Dataset 2**		**Dataset 3**	
	**WS**	**CS**	**LOSO**	**WS**	**LOSO**	**WS**	**LOSO**
*MS-EEGNet*	**83.52** **±** **1.67**	**86.38** **±** **1.60**	75.40 ± 1.81	**89.60** **±** **1.73**	74.82 ± 3.04	**92.63** **±** **1.77**	**86.09** **±** **1.88**
*EEGNet*	82.53 ± 1.83 ^**^	85.88 ± 1.63 ^**^	75.76 ± 1.71	87.98 ± 2.65	75.15 ± 3.01	91.22 ± 1.92 ^*^	83.30 ± 2.53
*BranchedNet*	77.43 ± 1.65 ^***^	84.20 ± 1.82 ^***^	**76.03** **±** **1.86**	83.34 ± 2.12 ^***^	72.39 ± 2.89	91.60 ± 1.53	84.84 ± 1.46
*OCLNN*	75.95 ± 1.64 ^***^	81.28 ± 1.65 ^***^	71.40 ± 1.42 ^**^	79.92 ± 2.78 ^***^	**75.21** **±** **3.14**	89.01 ± 2.03 ^***^	83.73 ± 1.59
*xDAWN+RG*	79.17 ± 1.43 ^***^	80.89 ± 1.32 ^***^	67.05 ± 1.71 ^**^	82.63 ± 2.07 ^***^	73.83 ± 2.71	90.03 ± 1.87 ^*^	82.40 ± 2.77

*The results of the performed Wilcoxon signed-rank tests (see section Statistics-i) are also reported (*

* *p < 0.05*,

** *p < 0.01*,

*** *p < 0.001, corrected for multiple tests). Within each column, the bold characters are used to denote the best performance among the tested algorithms*.

MS-EEGNet scored an AUC of 83.52 ± 1.67%, 89.6 ± 1.73%, and 92.63 ± 1.77% when using signals from datasets 1–3 adopting the WS strategy. The proposed architecture significantly outperformed all the tested SOA algorithms when using dataset 1, and significantly outperformed BranchedNet, OCLNN, and xDAWN+RG with dataset 2, and EEGNet, OCLNN, and xDAWN+RG with dataset 3. In addition, adopting the CS strategy, MS-EEGNet confirmed its decoding improvement with respect to the SOA, scoring an AUC of 86.38 ± 1.6%, outperforming significantly all the SOA algorithms. Lastly, adopting the LOSO strategy, MS-EEGNet scored an AUC of 75.4 ± 1.81%, 74.82 ± 3.04%, and 86.09 ± 1.88% when using signals from datasets 1–3. In this strategy, the proposed solution did not perform significantly better than the other SOA solutions (see section Performance of MS-EEGNet and Comparison With State-of-the-Art Algorithms) except for dataset 1 where MS-EEGNet outperformed OCLNN and xDAWN+RG.

#### Design Choices of MS-EEGNet

**Figure 2:**
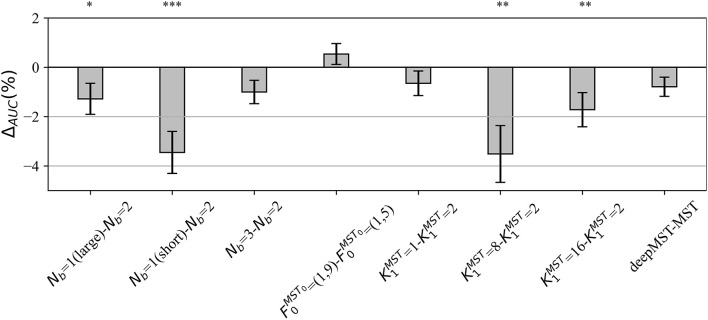
Impact of alternative design choices of MS-EEGNet on the performance metric. The figure reports the difference between the AUC scored with the variant and the baseline design (i.e., Δ_*AUC*_ = *AUC*_*variant*_−*AUC*_*baseline*_) for each condition of the hyper-parameter (*HP*) tested, reported on the x-axis as “*HP*_*variant*_−*HP*_*baseline*._” The height of each gray bar represents the mean value across the participants of Δ_AUC_, while the error bar (black lines) represents the standard error of the mean. The results of Wilcoxon signed-rank tests (see section Statistics-ii) are also reported (*p < 0.05, ** *p* < 0.01, *** *p* < 0.001, corrected for multiple tests) on top of the figure.

In the *post-hoc* hyper-parameter evaluation, we investigated the effect of particular design aspects of MS-EEGNet on the decoding performance, by statistically evaluating the difference in the AUCs between each variant MS-EEGNet and baseline MS-EEGNet (Δ_*AUC*_ = *AUC*_*variant*_−*AUC*_*baseline*_). The results are reported in Figure 2. In particular, the adoption of *N*_*b*_ = 1(large), *N*_*b*_ = 1(short), ${K_{1}}^{MST}=8$, ${K_{1}}^{MST}=16$ significantly worsened the performance, with an average drop in performance of 1.28, 3.46, 3.51, and 1.72%.

#### Variable Number of Training Examples: Within-Session and Transfer Learning Strategies

**Figure 3:**
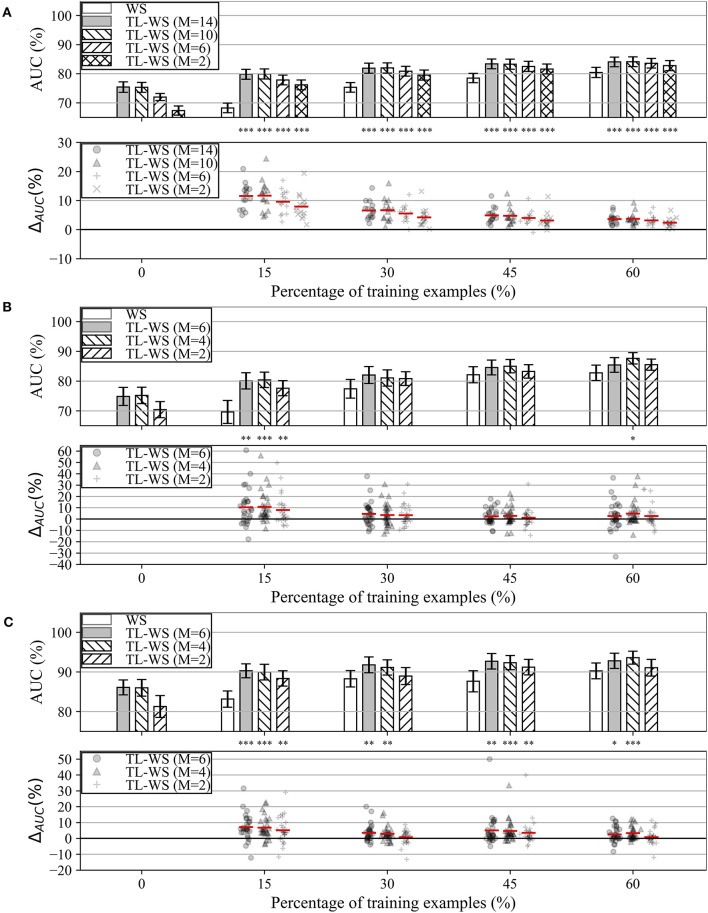
AUC obtained with MS-EEGNet trained with the WS and TL-WS strategies for datasets 1–3 (panels **A–C**, respectively). Top plot in each panel: The AUC obtained in WS (white bars) is reported as a function of the percentage of training examples (reported on the x-axis), while the AUC obtained in TL-WS is reported also as a function of the number of participants (*M*) used to optimize the LOSO-M models (gray and hatched bars). The height of each bar represents the mean value of the performance metric across the participants, while the error bar (black lines) represents the standard error of the mean. Bottom plot in each panel: The AUC difference between the TL-WS and WS strategies (i.e., Δ_*AUC*_ = *AUC*_*TL*−*WS*_−*AUC*_*WS*_) using the same percentage of training examples is reported using markers, and a red line denotes the mean value. For each percentage, a Wilcoxon signed-rank test was performed (see section Statistics-iii) to compare TL-WS vs. WS strategy, and the statistical significance is reported (*p < 0.05, **p < 0.01, ***p < 0.001, corrected for multiple tests) on top of each plot.

The performance obtained by MS-EEGNet in the WS strategy as a function of the percentage of training examples (reported on the x-axis) is reported in Figures 3A–C (white bars) for datasets 1–3. In all the datasets, a percentage of training trials of 30–45% was sufficient to obtain performance only a few points below that obtained with the entire training set, and in particular close or above 80%.

In addition, the performance obtained by MS-EEGNet in the TL-WS strategy is also reported as a function of: (i) the number of participants (*M*) adopted to design the LOSO-M model (gray and hatched bars); and (ii) the percentage of training examples. Lastly, the AUC difference between the TL-WS strategy and the WS strategy using the same percentage of training examples is shown in the lower panels of Figures 3A–C (Δ_*AUC*_ = *AUC*_*TL*−*WS*_−*AUC*_*WS*_).

In the case of dataset 1, the TL-WS strategy provided higher performance compared with the WS strategy (see the distributions of Δ_*AUC*_ reported in Figure 3) for each percentage of training examples ∀*M*. This occurred also in the case of dataset 3 except for a couple of conditions (*M* = 2 using 30 and 60% of the training examples of the held back participant). Using dataset 2, TL was found beneficial only with the lowest number of training examples (i.e., 15%) ∀*M* and using 60% of training examples with *M* = 4.

### Explaining P300 Decision: Gradient-Based Representations

In this section, we analyze the features of the input variables that most strongly supported the P300 classification decision in MS-EEGNet.

#### Spatio-Temporal Representations

**Figure 4:**
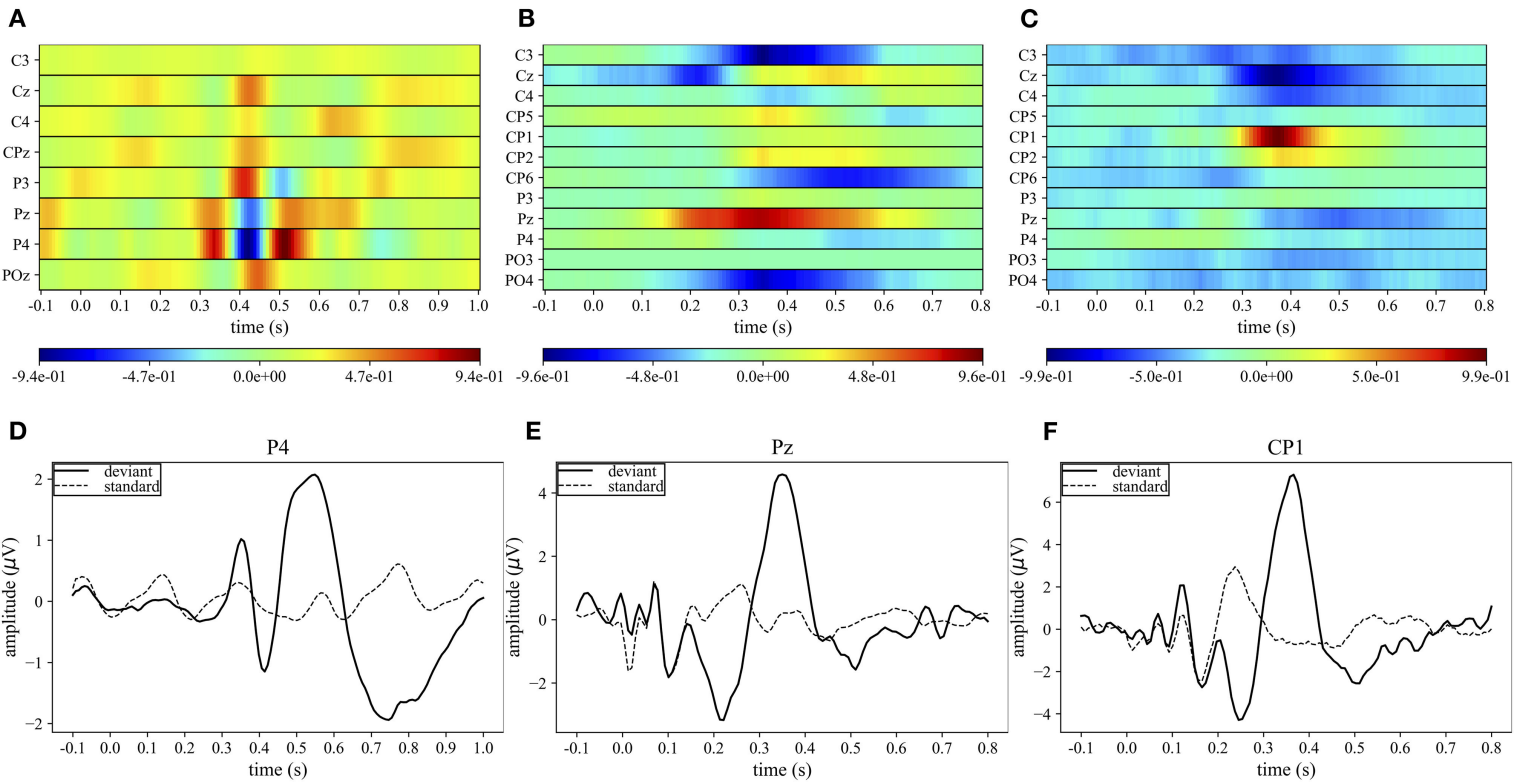
Grand average spatio-temporal representations. The top panels **(A–C)** show the grand average spatio-temporal representation of MS-EEGNet trained with the LOSO strategy using signals from datasets 1–3. Positive gradients are shown in red, while negative gradients are shown in blue. The bottom panels **(D–F)** show the grand average ERP for the deviant (black lines) and standard (dashed black lines) stimuli associated with the most relevant electrode (the one with the largest gradient values) for datasets 1–3.

Figures 4A–C display the grand average spatio-temporal representation of MS-EEGNet trained with the LOSO strategy using signals from datasets 1–3. From these figures, the more class-discriminative electrodes can be identified, i.e., P4, Pz, and CP1 for datasets 1–3, respectively. The grand average ERPs for the standard and deviant stimuli of these representative electrodes are displayed in Figures 4D–F.

In the case of dataset 1, P4 appeared as the most important electrode, in particular from 300 to 550 ms. Three main peaks can be identified: two positives at 350 and 510 ms, and a negative at ~410 ms (Figure 4A). These peaks correspond to the peaks in the grand average ERP of the deviant stimulus at approximately the same times (Figure 4D). In the cases of datasets 2 and 3, the most important sites were Pz from 300 to 400 ms and CP1 from 350 to 400 ms, respectively. In these cases, a single positive peak occurred in the spatio-temporal maps at about 350 and 390 ms, respectively (Figures 4B,C) and was associated with the peak in the grand average ERP of the deviant stimuli at approximately the same time (Figures 4E,F).

In the following sections, the interpretation of the relevant input features driving the MS-EEGNet P300 decision is analyzed separately in the temporal and spatial domains.

#### Absolute Temporal Representations

**Figure 5:**
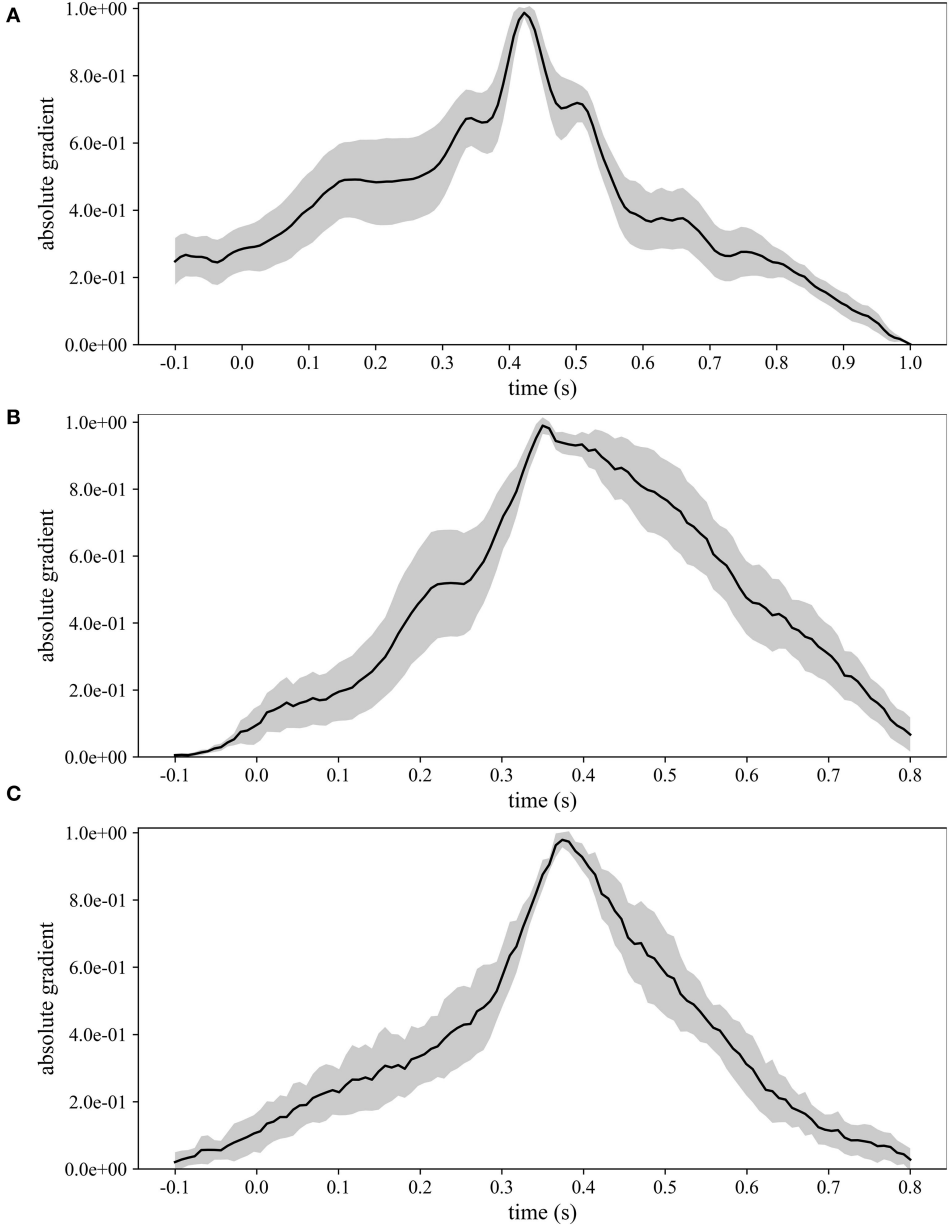
Grand average absolute temporal representations of MS-EEGNet trained with the LOSO strategy using signals from datasets 1–3 **(A–C)**; the mean value (black line) ± standard deviation (gray shaded areas) across participants are represented.

Figure 5 displays the grand average absolute temporal representations of MS-EEGNet trained with the LOSO strategy using signals from datasets 1–3 (Figures 5A–C). These patterns highlight, by means of local and global peaks, the more class-discriminative time samples for the P300 class across all spatial sites. These waveforms confirm the highest importance of time samples approximately between 300 and 550 ms in all the cases, with the peak at about 410, 350, and 390 ms for datasets 1–3, in agreement with the results shown in Figure 4. Interestingly, these waveforms synthetically highlight how the network learns different temporal profiles of sample relevance depending on the dataset, e.g., more regular waveforms in the cases of datasets 2 and 3 (but more spiking in the case of dataset 3) and more irregular waveforms in the case of dataset 1 (with several local maxima, two in particular just next to the global one, i.e., at 350 and 510 ms). These differences may be linked to the different sensory modalities involved (visual vs. auditory), different participants (healthy vs. pathological), or different paradigms used to elicit P300 (oddball paradigm vs. flashing the object under fixation).

#### Absolute Spatial Representations

**Figure 6:**
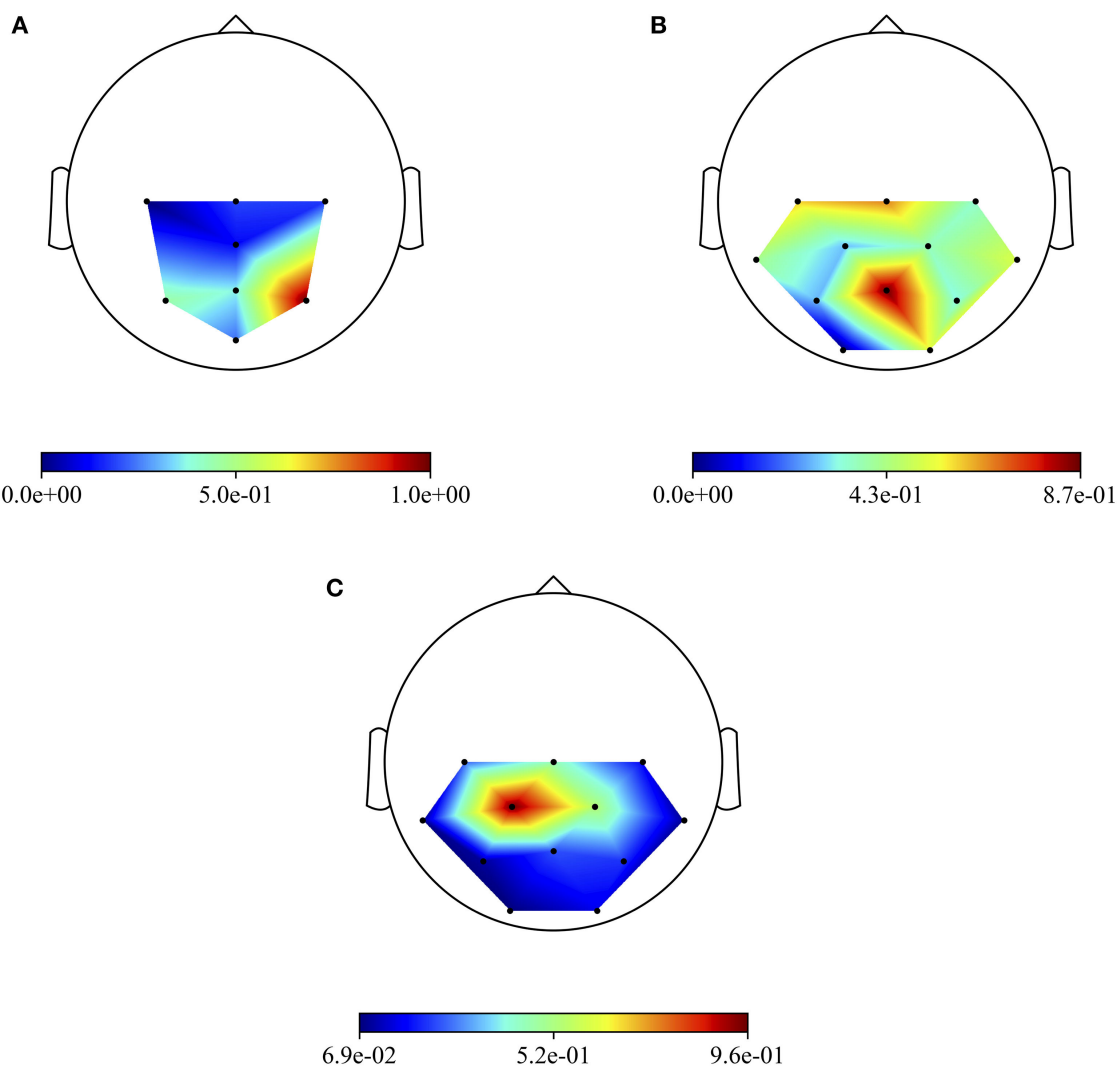
Grand average absolute spatial representations of MS-EEGNet trained with the LOSO strategy using signals from datasets 1–3 **(A–C)**.

Besides the investigation of the more P300-discriminative temporal features, it is also interesting to evidence the more P300-discriminative spatial features. To this aim, Figure 6 shows the grand average absolute spatial representations of MS-EEGNet trained with the LOSO strategy using signals from datasets 1–3 (Figures 6A–C), emphasizing the different spatial profiles of sample relevance.

The three more class-discriminative electrode sites across all the time samples were (in increasing order of relevance) Pz, P3, and P4 when the CNN was trained on dataset 1; C3, Cz, and Pz when the CNN was trained on dataset 2; and Cz, CP2, and CP1 when the CNN was trained on dataset 3. Again, these differences can be associated with differences in sensory modality, participants, and paradigms adopted across the three datasets.

#### Progressive Changes in Spatio-Temporal Sample Relevance While Increasing Training Examples

**Figure 7:**
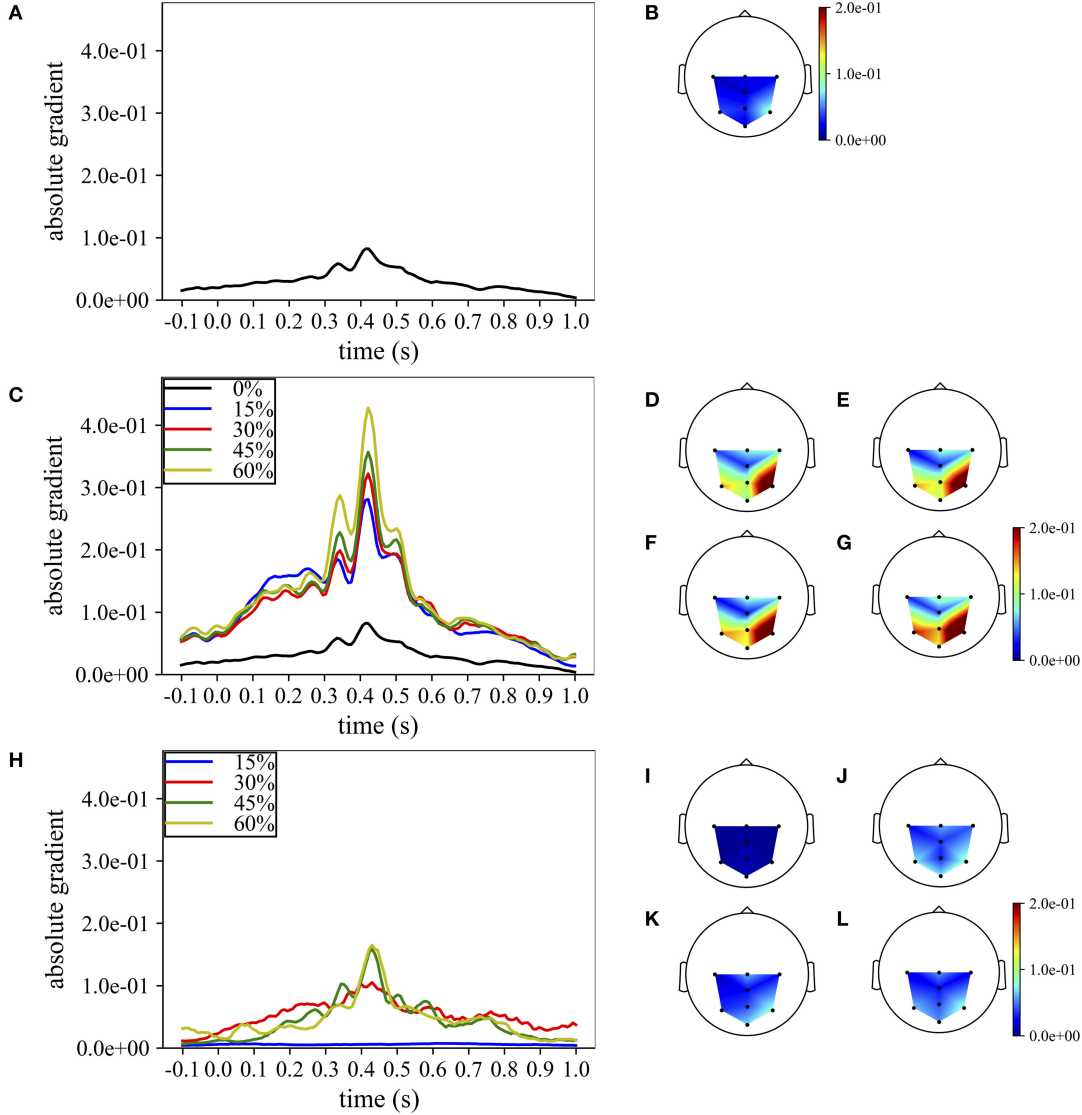
Grand average temporal and spatial absolute representations of MS-EEGNet trained on dataset 1 for a representative participant and session, adopting the LOSO, TL-WS, and WS strategies. In particular, the representations obtained using the LOSO strategy in the temporal and spatial domains are reported in **(A,B)**, respectively. The representations obtained using the TL-WS strategy in the temporal and spatial domains are reported in **(C)** (colored lines) and **(D–G)**, as the percentage of training examples of the new participant increased (15, 30, 45, 60%, from **D–G**). The representations obtained using the WS strategy in the temporal and spatial domains are reported in **(H)** (colored lines) and **(I–L)**, as the percentage of training examples of the participant increased (15, 30, 45, 60%, from **I–L**). Note that in order to maintain the same scale across the strategies in the spatial absolute representations, in **(D–G)**, the maximum gradient value represented (2.0e−1) was below the real maximum gradient value (3.3e−1), saturating the value in particular around P4.

Lastly, the absolute temporal and spatial representations were also used to analyze the progressive change in the importance of the spatio-temporal samples while increasing the percentage of training examples included when training MS-EEGNet with the TL-WS and WS strategies. For the TL-WS condition, only CNNs initialized from LOSO models with the largest number of participants were considered. The absolute temporal and spatial representations are reported in Figure 7, in case of a representative participant and session belonging to dataset 1.

In particular, Figures 7A,B report the absolute temporal and spatial representations as obtained in the LOSO strategy. Figures 7C–G show the effects of the TL-WS strategy, as the percentage of training examples from the held back participant increased. While transferring the knowledge from the other participants and sessions, the CNN inherited the importance profile from the pre-trained condition. Thus, for each percentage of training examples (Figures 7C–G), the temporal and spatial profiles did not change substantially their shape from the LOSO condition, since the importance in the temporal and spatial domains was already learned in the LOSO training. Nevertheless, the amplitude increased both in the temporal and spatial domains while increasing the percentage of training examples, indicating progressive accumulation of the importance. Conversely, adopting the WS strategy (Figures 7H–L), the CNN was randomly initialized and, therefore, had to learn from scratch the more class-discriminative spatio-temporal samples. Thus, the temporal and spatial profiles changed more with respect to TL-WS as the percentage of training examples increased. In particular, temporal profiles changed from a nearly flat profile (e.g., 15% in Figure 7H) to profiles more focused on time samples in the range of 300–550 ms (e.g., 45, 60% in Figure 7H) peaking at approximately 410 ms. Furthermore, the spatial profiles changed from a diffused distribution (Figure 7I) to distributions more focused on parietal electrodes (in particular P3, Pz, and P4 in Figures 7J–L). However, the absolute gradients resulted lower than in the TL-WS condition, in particular in correspondence of the more class-discriminative temporal (i.e., 350, 410, 510 ms) and spatial (P3, Pz, and P4) samples.

## Discussion

In this study, a lightweight multi-scale CNN design for EEG decoding named MS-EEGNet was proposed and applied to decode the P300 event from three different datasets. This CNN merges the multi-scale temporal learning proposed by Farahat et al. (2019) with lightweight characteristics originally proposed in EEGNet (Lawhern et al., 2018), operating even a further decrease in the number of trainable parameters while learning multi-scale features. MS-EEGNet was compared with many SOA algorithms, such as CNNs (EEGNet, BranchedNet, OCLNN) and a traditional ML pipeline (xDAWN+RG). To better analyze the multi-scale feature learning as operated by MS-EEGNet, we performed a *post-hoc* analysis on the hyper-parameters. In addition, MS-EEGNet was extensively evaluated under four training conditions, each one reflecting a different practical scenario: (i) using participant-specific signals of single recording sessions (WS); (ii) using participant-specific signals of multiple recording sessions (CS); (iii) using signals from the other participants (LOSO); and (iv) using a fraction of participant-specific signals from a pre-trained cross-participant CNN (TL-WS). Lastly, we exploited the saliency maps to obtain representations aimed to explain the MS-EEGNet decision by visualizing the relevant samples in the input domain. Both the proposed architecture and the performed analyses represent significant expansion compared with the previous study (Borra et al., 2020a), limited to the application of a design based on EEGNet to solve the P300 task proposed by the IFMBE 2019 scientific challenge (corresponding to dataset 1 here). In the following, the performance of MS-EEGNet and the results of the performed analyses are critically discussed.

### Performance of MS-EEGNet and Comparison With State-of-the-Art Algorithms

The performance of MS-EEGNet using the WS strategy was above 80% for all the datasets, reaching higher values for datasets 2 and 3 compared with dataset 1 (Table 3). This difference could depend on several factors, such as different paradigms, stimuli, and populations (ASD vs. healthy), possibly leading to different P300 responses, e.g., with lower or higher amplitude. Regarding this, Figures 4D–F show that the P300 response to the deviant stimulus in dataset 1 was indeed characterized by a lower amplitude, perhaps increasing the difficulty in discriminating between standard/deviant stimuli. Other contributing factors could be the lower proportion between training and test examples, and the lower number of electrodes in dataset 1 vs. datasets 2 and 3. It is worth noticing that this same difference in the WS performance across the datasets was notable in the other algorithms, too. Using the CS strategy, the performance improved compared with the WS strategy for all the algorithms, and this result is in line with Simões et al. (2020). When comparing MS-EEGNet to the other algorithms, the design exhibited the highest performance on each dataset, adopting the WS and CS strategies. Interestingly, among the tested CNNs, OCLNN (which uses a mixed spatio-temporal convolution) and BranchedNet (which performs a spatial convolution first) performed generally lower than MS-EEGNet and EEGNet (which perform temporal convolution first). This is in line with Simões et al. (2020), where the previous design adapted from EEGNet outperformed significantly a CNN design inspired by Manor and Geva (2015) that used a first spatial convolutional layer. Therefore, these results suggest that a CNN design trained on participant-specific signals and based on a first temporal filtering of EEG signals leads to higher P300 decoding performance than other solutions that use first mixed spatio-temporal or first spatial filtering of the input signals. Hence, higher performance could be achieved learning temporal features directly from raw EEG signals (exploiting useful raw temporal information related to the P300 event) instead from signals with a higher level of abstraction. Overall, among the tested SOA CNNs, EEGNet is the one exhibiting the closest performance to MS-EEGNet; and this can be explained by the derivation of MS-EEGNet from EEGNet with the addition of multi-scale temporal feature learning and compressed representation learning. However, the results denote that the changes included in MS-EEGNet can significantly improve the high performance already achieved by EEGNet, especially using session-specific (WS) and participant-specific (CS) input distributions, see datasets 1 (*p* = 2e−3 and *p* = 3e−3 with the WS and CS strategies, respectively) and 3 (*p* = 4e−2) in Table 3, using a lower number of trainable parameters.

As expected, adopting the LOSO strategy caused an overall drop of the performance metric across all the tested approaches, with respect to the WS and CS strategies; and the different approaches generally provided similar performance (MS-EEGNet only performed significantly better than xDAWN+RG and OCLNN in dataset 1).

Hence, overall, MS-EEGNet performed better than the other SOA algorithms in the WS and CS strategies and behaved similarly with the other SOA algorithms in the LOSO strategy. This becomes more relevant considering that MS-EEGNet is the lightest CNN among the tested ones, as EEGNet, BranchedNet, and OCLNN introduced more trainable parameters (see Table 2). Indeed, this is particularly important, as in practice it is common to deal with small EEG datasets. Thus, keeping limited the number of trainable parameters is crucial when designing CNNs for EEG decoding in order to avoid overfitting. Likely, the lightweight design of MS-EEGNet may explain the absence of higher performance in the LOSO strategy due to the peculiarities of the LOSO training. In this case, class-discriminative features are learned from input distributions with very large variability, involving different participants and possibly different sessions (e.g., with dataset 1). Thus, the CNN, besides needing more training examples, may need more capacity (i.e., more layers/more parameters) to solve the task with higher performance. Considering that the CNN is the lightest among the tested ones (see Table 2), obtaining performance similar with that of the other CNNs should not be surprising (and rather can be still considered a satisfactory result). In the LOSO strategy, MS-EEGNet significantly outperformed the traditional ML approach only for dataset 1. This may indicate that in the LOSO strategy MS-EEGNet can learn more relevant cross-participant features, leading to significant higher performance, than an ML pipeline when a larger dataset is used, as in the case of dataset 1. Lastly, besides performance and parameters to fit, considerations about the training time are relevant for practical usage. The multi-scale SOA CNN (BranchedNet) was slower to train with respect to MS-EEGNet, while single-scale SOA CNNs (EEGNet and OCLNN) were faster to train. Overall, compared with SOA CNNs, MS-EEGNet represented a good compromise between performance, model size and computational time.

### Performance of MS-EEGNet: *post-hoc* Hyper-Parameter Evaluation

We performed a *post-hoc* hyper-parameter evaluation of eight variant design choices of MS-EEGNet by varying four different hyper-parameters of the multi-scale temporal block (Figure 2). Using a single-scale variant (*N*_*b*_ = 1) that includes only the large or the short scale, a reduction in trainable parameters and in training time was observed with respect to the baseline MS-EEGNet (see Table 2). At the same time, the performance significantly worsened in both cases, indicating the benefit of the multi-scale temporal feature learning with respect to single-scale feature learning for P300 decoding, at the expense of an increased number of trainable parameters and computational time. In addition, the different impact on the performance observed in the design *N*_*b*_ = 1 (large) and *N*_*b*_ = 1 (short) suggests that the temporal features learned in the large-scale branch were more class-discriminative. Interestingly, using an additional intermediate timescale (three-branched variant *N*_*b*_ = 3), a non-significant difference in performance was observed compared with the baseline MS-EEGNet, while more parameters and training time were required (see Table 2). These results about the number of branches of MS-EEGNet suggest that the dual-branched design represented good compromise between performance, model size, and training time.

Furthermore, alternative ratio $r^{MST}=\frac{1}{2}$ between the two timescales obtained with ${F_{0}}^{MST_{0}}=\left(1,9\right)$ (corresponding to learning summaries of about 500 and 250 ms), resulted in a small, not significant (*p* = 0.06) increase in performance with respect to the baseline MS-EEGNet ($r^{MST}=\frac{1}{4}$), requiring few more parameters and training time. In addition, variants learning more feature maps (${K_{1}}^{MST}=8$ and ${K_{1}}^{MST}=16$), with respect to the compressed representation exploited in the baseline MS-EEGNet (${K_{1}}^{MST}=2$) not only required more parameters to fit and were slower (see Table 2) but worsened the performance significantly. This suggests that learning compressed representations could be beneficial in terms of performance, model size, and training time for P300 decoding. Remarkably, the variant architecture including the most extreme compressed representation (${K_{1}}^{MST}=1$), i.e., learning only a feature map for each timescale, scored similar performance as the baseline MS-EEGNet while lightly reducing the model size and requiring the same training time (see Table 2), suggesting that future architectures could also exploit this design to further reduce the model size without hampering the performance. Lastly, increasing the depth of the MST block did not provide any significant improvement in performance, introduced more parameters to fit, and required more training time (see Table 2). Thus, these last results suggest that a shallower and lightweight MST design, as provided in the baseline MS-EEGNet, is preferable for P300 decoding.

### Performance of MS-EEGNet: Transfer Learning Strategy and Variable Number of Training Trials

MS-EEGNet was capable to deal with a reduced number of training trials when trained from scratch (WS), although not at the smallest percentage of training trials (Figure 3). The performance increased in TL-WS. Indeed, transferring knowledge using the smallest percentage of training examples of the held back participant (i.e., 15%) resulted in a beneficial effect, compared with WS across all the datasets and regardless of the number of participants from whom the knowledge was transferred (Figure 3). This beneficial effect of the TL-WS strategy was also found when using more training examples (30, 45, and 60%) of the held back participant on datasets 1 and 3. As expected, the worst performance was obtained when transferring knowledge from the LOSO models trained on the smallest subset of participants (*M* = 2) for all datasets and percentages. However, this condition produced a significant increase in performance compared with randomly initialized models especially when using a small number of signals belonging to the new user (i.e., 15%). Therefore, pre-trained models do not necessarily need to be optimized on a large set of participants in order to significantly outperform randomly initialized models, especially when using a small amount of data during transfer learning (see also section 3 in Supplementary Materials for comparison between TL-WS and WS with 100% of training trials).

Overall, these results suggest that the proposed approach could be used to accurately decode the P300 event even with a reduced number of standard/deviant stimuli presented to the user during the calibration stage.

### Explaining P300 Decision

The proposed approach achieved high performance, outperforming the SOA algorithms. As stated by Montavon et al. (2018), in practice it is also crucial to verify that the decoding performance results from a proper problem representation and not from the exploitation of artifacts in the input data. Therefore, in this study, we explained the MS-EEGNet decision for P300 decoding *via* the saliency maps, providing GA spatio-temporal, GA absolute temporal, and GA absolute spatial representations of the relevance of the input samples.

The GA spatio-temporal representations of MS-EEGNet (Figures 4A–C) evidenced higher values (both positive and negative) of the gradients, corresponding to more class-discriminative input samples, within time intervals (roughly between 300 and 550 ms) matching the P300 temporal occurrence for all the datasets. The positive/negative peaks in these gradient patterns corresponded to peaks in the GA ERPs of the deviant stimulus (Figures 4D–F). Indicating with i and j are the row and column indices, respectively; and the positive and negative gradients in the (i, j) location shown in Figures 4A–C represent the direction in which change in the (i,j) input feature increased the P300 class score and, consequently, the CNN decision toward the P300 class. Thus, for example, analyzing the gradients related to P4 obtained from dataset 1 (Figure 4A), two positive peaks and a negative peak were found. As the P4 input signal of a deviant trial increased its value at the two positive peaks (at about 350 and 510 ms), the deviant condition differed more than the standard condition, resulting in the deviant class being easier to distinguish and providing a higher score to it. Therefore, these peaks in the deviant GA ERP were associated with positive gradient peaks. Conversely, as the P4 input signal of a deviant trial reduced its value at the local minimum (at ~410 ms), the negative peak resulted more distant from the standard condition, leading to a higher score for the deviant class (negative gradient peak). This consideration can be extended to datasets 2 and 3, by analyzing the Pz and CP1 electrodes. Therefore, as already obtained in Farahat et al. (2019), higher differences in the ERP between deviant and standard stimuli are reflected onto the saliency maps by means of positive and negative gradients.

When computing the absolute value of the saliency maps, the absolute gradient at the spatio-temporal sample (i,j) reflects how much a change in this sample affects the P300 class score. We analyzed the absolute saliency maps separately in the time and spatial domains (Figures 5, 6) in order to evidence the more discriminative temporal samples and electrodes independently on the direction (positive or negative) they contributed to the decoding result. The GA temporal absolute profile for each dataset peaked approximately in correspondence with the peak of the P300 response. Interestingly, the absolute temporal representations exhibit different patterns for the three datasets, evidencing that they are able to detect differences embedded in the P300 response across the three datasets. Lastly, the GA absolute spatial distributions represented in a topological map allowed a direct analysis of the more P300 discriminative electrodes of MS-EEGNet. These were mainly distributed in the parietal and centro-parietal areas. This may provide practical hints to reduce the number of electrodes in the design of P300-BCIs. Overall, the various gradient-based representations (Figures 4–6) matched the P300 spatio-temporal distribution, confirming that MS-EEGNet was able to capture meaningful task-related features, without exploiting artifactual/noisy input sources.

Interestingly, using a representative example, we show that while transferring knowledge the importance of temporal and spatial samples gradually increased from the LOSO condition (Figures 7A,B) as the percentage of training examples increased. In particular, it appears that the more task-relevant temporal and spatial samples were already learned in the LOSO strategy. However, during transfer learning (Figures 7C–G), the LOSO temporal and spatial profiles (template profiles) were modeled on the new participant- and session-specific training distribution, giving progressively more importance to particular temporal intervals/electrode sites starting from the template profiles. The availability of these template profiles allowed rapid learning of the relevant participant-specific and session-specific input samples (i.e., needing a low number of training examples of the new participant). Conversely, when training CNNs from scratch with the WS strategy, the profile distribution rapidly changed its shape both in the temporal (Figure 7H) and spatial (Figures 7I–L) domains but reached lower importance values compared with the TL-WS strategy. When transferring knowledge, the profile was more focused on interval 300–550 ms with three distinct main peaks and on sites P4 > P3 > Pz already at the lowest percentage (15%, Figures 7C,D); while at the same percentage, the WS strategy was characterized by more flat and homogeneous distributions (Figures 7H,I). These considerations could explain the performance improvement obtained in the TL-WS strategy (Figure 3): the parameters learned using the LOSO strategy overall represented a better initialization point in the parameter space compared with a random one.

## Concluding Remarks

In conclusion, we wish to stress that this study aims to contribute to uncovering the enormous potentialities of deep learning via CNNs for EEG decoding and to their exploitation in practice adopting different training strategies, reflecting different scenarios. The multi-scale design was the most lightweight and at the same time outperformed many SOA algorithms when using three different P300 datasets, indicating that care has to be taken to design CNNs for EEG decoding, keeping limited the parameters to fit, especially when handling small datasets (not as large as the ones adopted in the computer vision field, e.g., > 100 K of examples). In addition, the hyper-parameter *post-hoc* analysis confirmed that the innovative aspects of the architecture, i.e., the design of a lightweight multi-scale temporal block implemented *via* separable convolutions and the use of compressed representation learning were beneficial. Crucially, the capability of MS-EEGNet to transfer knowledge with high performance even with a small number of training examples could be highly useful in practice to reduce the calibration time of P300-based BCIs on a new user.

Saliency maps confirmed their utility to explain the neural network decision in P300 decoding tasks; the derived spatial and temporal representations resulted to match the P300 spatio-temporal distribution. However, the utility of these representations is not limited to provide an additional validation of the algorithm. Indeed, the CNN ability to learn automatically the most meaningful features to perform classification gives the possibility to use these algorithms as data-driven EEG analysis tools. Then, the use of the saliency maps (or similar representations) allows the interpretation of the CNN decision, and it is possible to take advantage of these interpretations for increasing the comprehension of brain dynamics underlying decoded events (e.g., P300 response). For example, representations derived from saliency maps (in the time and/or spatial domain) could be used to study the variability between participants (i.e., which features of the input samples are more/less consistent across participants) and within-participant (i.e., by comparing representations associated with early and late trials, e.g., to investigate the effects of training or treatment). Furthermore, the analysis of between-participants and within-participant variabilities could be useful, in perspective, to develop biomarkers to diagnose and monitor neurological or psychiatric disorders (Farahat et al., 2019), e.g., P300 amplitude, latency, and topographical alterations in mild cognitive impairment (Medvidovic et al., 2013), dementia (Vecchio and Määttä, 2011), and schizophrenia (Jeon and Polich, 2003). In addition, identifying the more class-discriminative temporal and spatial input features can also have a relevant practical impact on the design of BCIs. For example, the identification of a small subset of more relevant electrodes (as we found here) may drive the definition of BCI systems with a very small electrode montage, increasing the comfort of a participant and reducing preparation time. It is worth noticing that by performing this analysis on within-participant CNNs, the optimal electrode montage could also be identified on an individual basis.

Overall, this study, by specifically addressing the aspects of lightweight design, transfer learning, and interpretability of the proposed CNN, can contribute to advance the development of deep learning-based decoders for P300-BCIs. Future developments include the application of the proposed architecture to other ERP decoding tasks, and the adoption of interpretable and more lightweight layers, such as the sinc-convolutional layer, to perform band-pass filtering (Ravanelli and Bengio, 2018; Borra et al., 2020b,c). In addition, automatic hyper-parameter search (Snoek et al., 2012) will be exploited to further improve the MS-EEGNet design and other explanation techniques, such as layer-wise relevance propagation, will be investigated, carefully analyzing the effect of different propagation rules and parameters for EEG decoding.

## Data Availability Statement

The dataset 1 used in this study can be found online at https://www.kaggle.com/disbeat/bciaut-p300. The datasets 2 and 3 used in this study are available under request to the corresponding author DB. Codes are available at https://github.com/ddavidebb/P300_decoding_MS-EEGNet.

## Ethics Statement

The studies involving human participants were reviewed and approved by Bioethics Committee, University of Bologna (datasets 2 and 3). The patients/participants provided their written informed consent to participate in this study.

## Author Contributions

DB and EM conceived and designed the methodology and wrote the original draft. EM designed the recording protocol for datasets 2 and 3 and contributed to acquiring signals. DB processed data. DB, SF, and EM critically analyzed the data and reviewed and edited the manuscript. All authors contributed to the article and approved the submitted version.

## Conflict of Interest

The authors declare that this study received materials from NVIDIA Corporation with the donation of the TITAN V used for this research. The provider was not involved in the study design, collection, analysis, interpretation of data, the writing of this article or the decision to submit it for publication.
