# Supplementary material for: A Lightweight Multi-Scale Convolutional Neural Network for P300 Decoding: Analysis of Training Strategies and Uncovering of Network Decision
Source: Front Hum Neurosci. 2021 Jul 8;15:655840. doi: 10.3389/fnhum.2021.655840 (PMC8295472; doi:10.3389/fnhum.2021.655840)
Supplement: Supplementary file 1 [file Data_Sheet_1.pdf]

SUPPLEMENTARY MATERIAL

1. Details about the state-of-the-art CNNs

The main details of the architectures considered in this study are reported in the Supplementary Tables 1-3, respectively for our adaptation of EEGNet that won the IFMBE 2019 competition (Borra et al., 2020a) (code and documentation available at https://github.com/ddavidebb/IFMBE2019Challenge-BCIAUT-P300), BranchedNet (Farahat et al., 2019) and OCLNN (Shan et al., 2018). Since BranchedNet was designed to accept EEG signals sampled at 250 Hz as input, we changed the temporal pool sizes and kernel lengths accordingly (i.e. dividing them with a factor of 2), as done in previous studies when re-implementing CNNs on other datasets (Lawhern et al., 2018; Borra et al., 2020c). Codes of all the re-implemented CNNs are available together with the ones of MS-EEGNet at https://github.com/ddavidebb/P300_decoding_MS-EEGNet.

2. Details about the state-of-the-art traditional machine learning pipeline

The traditional machine learning pipeline was inspired from the re-implementation of Lawhern et al. (2018) of the approach that won the BCI challenge (https://www.kaggle.com/c/inria-bci-challenge) proposed as part of the IEEE Neural Engineering Conference 2015 (code and documentation available at http://github.com/alexandrebarachant/bci-challenge-ner-2015). In particular, our pipeline consisted of 4 main steps:

1. Learn two sets (one for the non-P300 class and one for the P300 classes) of 5 xDAWN spatial filters (Rivet et al., 2009), using the ERP template concatenation method described in (Barachant and Congedo, 2014).
2. Project the covariance matrices onto the tangent space using the log-euclidean metric (Barachant et al., 2012).
3. Feature normalization using a L_1 ratio of 0.5 (equal weight for L_1 and L_2 penalties).
4. Classification using Elastic Net regression using an α parameter (constant that multiplies the penalty terms) of $2 \cdot 10^{-4}$.

The pipeline was maintained the same for the three datasets, except for the number of input channels (8 for the dataset 1, 12 for datasets 2 and 3).

3. Comparison between transferring the knowledge on a small dataset and learning from scratch on the entire dataset

The main text shows the comparison between the TL-WS and the WS strategy (see Figure 3) using the same percentage of training examples and number of participants from whom the knowledge was transferred (M). Here, the TL-WS strategy was also compared to the WS strategy trained on 100% of training examples of the new user (corresponding to the baseline WS strategy, see Section 2.3-i of the manuscript). This test was performed in order to compare the results obtained while transferring the knowledge (as a function of the percentage of training examples and M) with the traditional WS strategy that used the entire training set. The statistical analysis adopted for this test is the same as the one used in Section 2.5-iii of the manuscript.

In Supplementary Figure 1, the difference between the AUC scored with the TL-WS strategy and the traditional WS strategy (i.e. using 100% of training examples, "$WS100$") is reported ($\Delta_{AUC}= AUC_{TL-WS} - AUC_{WS100}$). The performance was comparable or even significantly higher (see $M = 10$ using 60% of examples) from 45% of training examples for dataset 1, especially for higher M values. For dataset 3, the performance was comparable for each percentage in case of the highest M value ($M = 6$), from 30% in case of $M = 4$ and for 60% in case of $M = 2$. Lastly, for dataset 2, no significant difference between the two trainings strategies was obtained only when using 60% of training examples and $M = 4$ in the TL-WS strategy.

SUPPLEMENTARY FIGURES AND TABLES

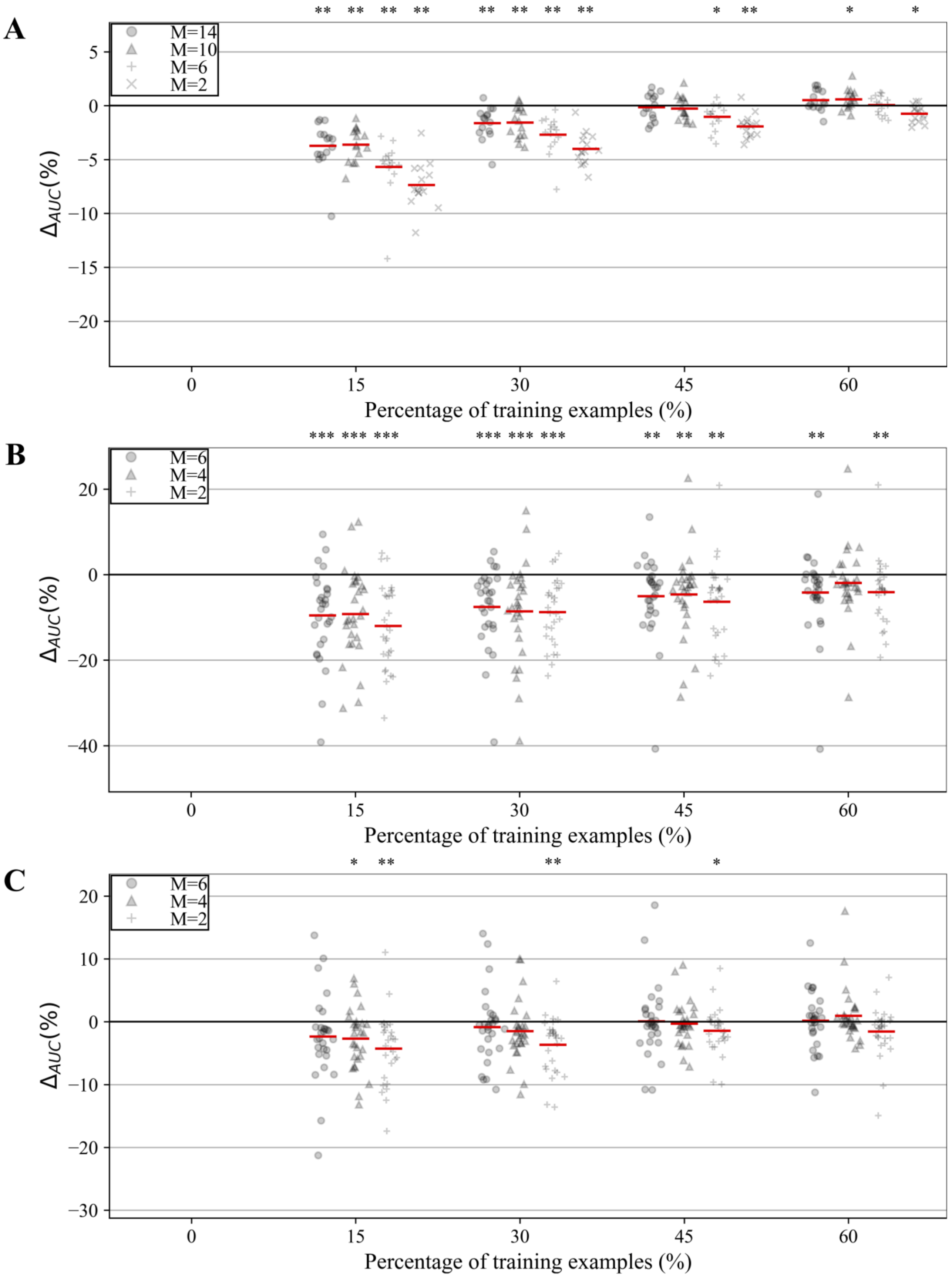

Supplementary Figure 1 – AUC difference between MS-EEGNet trained with the TL-WS and WS strategy using the entire training set (i.e. $\Delta_{AUC} = AUC_{TL-WS} - AUC_{WS100}$) for datasets 1-3 (Supplementary Figures 1A-C). The AUC difference is reported using markers and a red line denoting the mean value, as a function of the number of participants (M) used to optimize the LOSO-M models and of the training examples of the new user (reported on the x-axis). For each percentage, a Wilcoxon signed-rank test was performed to compare TL-WS vs. WS strategy, and the statistical significance is reported (*p<0.05, **p<0.01, ***p<0.001, corrected for multiple tests) on top of each plot.

Supplementary Table 1 – Architecture details of the EEGNet adaptation used in (Borra et al., 2020a). Each layer is provided with its name, main hyper-parameters and number of trainable parameters. See Sections 2.1 and 2.2 of the manuscript for the meaning of the symbols. The total number of trainable parameters was 1386, when using signals from the dataset 1, and 1418, when using signals from datasets 2 and 3. In all layers, where not specified, stride (S) and padding (P) were set to $(1,1)$ and $(0,0)$, respectively.

Block	Layer name	Hyper-parameters	Number of trainable parameters
	Input	$K_0 = 1$	0
ST	Conv2D	$K_0{}^{\mathrm{ST}} = 8$, $F_0{}^{\mathrm{ST}} = (1{,}65)$, $P_0{}^{\mathrm{ST}} = (0{,}32)$	$F_0{}^{\mathrm{ST}}[0] \cdot F_0{}^{\mathrm{ST}}[1] \cdot K_0{}^{\mathrm{ST}} \cdot K_0$
	BatchNorm2D	$m = 0.99$	$2 \cdot K_0{}^{\mathrm{ST}}$
	Depthwise-Conv2D	$D_1{}^{\mathrm{ST}} = 2$, $K_1{}^{\mathrm{ST}} = K_0{}^{\mathrm{ST}} \cdot D_1{}^{\mathrm{ST}}$, $F_1{}^{\mathrm{ST}} = (C, 1)$, kernel max norm=1	$F_1{}^{\mathrm{ST}}[0] \cdot F_1{}^{\mathrm{ST}}[1] \cdot K_1{}^{\mathrm{ST}}$
	BatchNorm2D	$m = 0.99$	$2 \cdot K_1{}^{\mathrm{ST}}$
	ELU	$\alpha = 1$	0
	AvgPool2D	$F_p{}^{\mathrm{ST}} = S_p{}^{\mathrm{ST}} = (1{,}4)$	0
	Dropout	$p = 0.25$ or $p = 0.5$	0
Temporal (T)	Separable-Conv2D	$D_0{}^T = 1, K_0{}^T = K_1{}^{ST} \cdot D_0{}^T$, $F_0{}^T = (1{,}17)$, $P_0{}^T = (0{,}8)$	$F_0{}^T[0] \cdot F_0{}^T[1] \cdot K_0{}^T + \left(K_0{}^T\right)^2$
	BatchNorm2D	$m = 0.99$	$2 \cdot K_0{}^T$
	ELU	$\alpha = 1$	0
	AvgPool2D	$F_p{}^T = S_p{}^T = (1{,}8)$	0
	Dropout	$p = 0.25$ or $p = 0.5$	0
FC	Flatten		0
	Fully-Connected	$N^{FC} = 2$, kernel max norm=0.25	$N^{FC} \cdot (T_p{}^T \cdot K_0{}^T + 1)$
	Softmax		0

Supplementary Table 2 – Architecture details of BranchedNet (Farahat et al., 2019). Each layer is provided with its name, main hyper-parameters and number of trainable parameters. See Sections 2.1 and 2.2 of the manuscript for the meaning of the symbols. The total number of trainable parameters was 5418, when using signals from the dataset 1, and 7954, when using signals from datasets 2 and 3. In all layers, where not specified, stride (S) and padding (P) were set to (1,1) and (0,0), respectively.

Block	Layer name	Hyper-parameters	Number of trainable parameters
	Input	$K_0 = 1$	0
Spatial (S)	Conv2D	$K_0{}^S = 12$, $F_0{}^S = (C, 1)$	$F_0{}^S[0] \cdot F_0{}^S[1] \cdot K_0{}^S \cdot K_0$
	BatchNorm2D	$m = 0.99$	$2 \cdot K_0{}^S$
	Tanh		0
	Dropout	$p = 0.25$ or $p = 0.5$	0
MST scale 0	Conv2D	$K_0{}^{MST_0} = 4$, $F_0{}^{MST_0} = (1,12)$	$F_0{}^{MST_0}[0] \cdot F_0{}^{MST_0}[1] \cdot K_0{}^{MST_0} \cdot K_0{}^S$
	BatchNorm2D	$m = 0.99$	$2 \cdot K_0{}^{MST_0}$
	Tanh		0
	Dropout	$p = 0.25$ or $p = 0.5$	0
	AvgPool2D	$F_p{}^{MST_0} = S_p{}^{MST_0} = (1,2)$	0
	Conv2D	$K_1{}^{MST_0} = 8$, $F_1{}^{MST_0} = (1,12)$	$F_1{}^{MST_0}[0] \cdot F_1{}^{MST_0}[1] \cdot K_1{}^{MST_0} \cdot K_0{}^{MST_0}$
	BatchNorm2D	$m = 0.99$	$2 \cdot K_1{}^{MST_0}$
	Tanh		0
	Dropout	$p = 0.25$ or $p = 0.5$	0
	Conv2D	$K_2{}^{MST_0} = 8$, $F_2{}^{MST_0} = (1,12)$	$F_2{}^{MST_0}[0] \cdot F_2{}^{MST_0}[1] \cdot K_2{}^{MST_0} \cdot K_1{}^{MST_0}$
	BatchNorm2D	$m = 0.99$	$2 \cdot K_2{}^{MST_0}$
	Tanh		0
	Dropout	$p = 0.25$ or $p = 0.5$	0
	Flatten		0
MST scale 1	Conv2D	$K_0{}^{MST_1} = 4$, $F_0{}^{MST_1} = (1,52)$	$F_0{}^{MST_1}[0] \cdot F_0{}^{MST_1}[1] \cdot K_0{}^{MST_1} \cdot K_0{}^S$
	BatchNorm2D	$m = 0.99$	$2 \cdot K_0{}^{MST_1}$
	Tanh		0
	Dropout	$p = 0.25$ or $p = 0.5$	0
	AvgPool2D	$F_p{}^{MST_1} = S_p{}^{MST_1} = (1,2)$	0
	Flatten		0
FC	Concatenate		0
	Fully-Connected	$N^{FC} = 2$	$N^{FC} \cdot (T_2{}^{MST_0} \cdot K_2{}^{MST_0} + T_p{}^{MST_1} \cdot K_0{}^{MST_1} + 1)$
	Softmax		0

Supplementary Table 3 – Architecture details of OCLNN (Shan et al., 2018). Each layer is provided with its name, main hyper-parameters and number of trainable parameters. See Sections 2.1 and 2.2 of the manuscript for the meaning of the symbols. The total number of trainable parameters was 1650, when using signals from the dataset 1, and 1874, when using signals from datasets 2 and 3. In all layers, where not specified, stride (S) and padding (P) were set to $(1,1)$ and $(0,0)$, respectively.

Block	Layer name	Hyper-parameters	Number of trainable parameters
	Input	$K_0 = 1$	0
Mixed ST (mST)	Conv2D	${K_0}^{\text{mST}} = 16$, ${F_0}^{\text{mST}} = (C, 9)$, ${S_0}^{\text{mST}} = (1,9)$	${F_0}^{\text{mST}}[0] \cdot {F_0}^{\text{mST}}[1] \cdot {K_0}^{\text{mST}} \cdot K_0 + {K_0}^{\text{mST}}$
	ReLU		0
	Dropout	$p = 0.25$ or $p = 0.5$	0
FC	Flatten		0
	Fully-Connected	$N^{FC} = 2$	$N^{FC} \cdot ({T_0}^{\text{mST}} \cdot {K_0}^{\text{mST}} + 1)$
	Softmax		0
